# Faecal neutrophil elastase-antiprotease balance reflects colitis severity

**DOI:** 10.1038/s41385-019-0235-4

**Published:** 2019-11-26

**Authors:** Rachael Barry, David Ruano-Gallego, Shiva T Radhakrishnan, Scott Lovell, Lu Yu, Olga Kotik, Izabela Glegola-Madejska, Edward W Tate, Jyoti S Choudhary, Horace R T Williams, Gad Frankel

**Affiliations:** 1 Department of Life Sciences, MRC Centre for Molecular Bacteriology and Infection, Imperial College London, London, UK; 2 Department of Gastroenterology and Hepatology, St Mary’s Hospital, Imperial College Healthcare NHS Trust, London, UK; 3 Department of Metabolism, Digestion and Reproduction, Faculty of Medicine, Imperial College London, London, UK; 4 Department of Chemistry, Imperial College London, London, UK; 5 Chester Beatty Laboratories, Institute of Cancer Research, Mary-Jean Mitchell Green Building, 237 Fulham Road, London, UK

## Abstract

Given the global burden of diarrheal diseases on healthcare it is surprising how little is known about the drivers of disease severity. Colitis caused by infection and inflammatory bowel disease (IBD) is characterised by neutrophil infiltration into the intestinal mucosa and yet our understanding of neutrophil responses during colitis is incomplete. Using infectious (*Citrobacter rodentium*) and chemical (dextran sulphate sodium; DSS) murine colitis models, as well as human IBD samples, we find that faecal neutrophil elastase (NE) activity reflects disease severity. During *C. rodentium* infection intestinal epithelial cells secrete the serine protease inhibitor SerpinA3N to inhibit and mitigate tissue damage caused by extracellular NE. Mice suffering from severe infection produce insufficient SerpinA3N to control excessive NE activity. This activity contributes to colitis severity as infection of these mice with a recombinant *C. rodentium* strain producing and secreting SerpinA3N reduces tissue damage. Thus, uncontrolled luminal NE activity is involved in severe colitis. Taken together, our findings suggest that NE activity could be a useful faecal biomarker for assessing disease severity as well as therapeutic target for both infectious and chronic inflammatory colitis.

## Introduction

Inflammatory diarrhoeal diseases are major health concerns worldwide. Inflammatory diarrhoea can result from infections with enteric pathogens or chronic illnesses, such as inflammatory bowel diseases (IBDs), including Crohn’s disease (CD) and ulcerative colitis (UC).^1^ Although the cause is different, disease pathology is similar, including dysbiosis, disruption of the mucosal barrier and extensive inflammation. Mild cases of colitis can be managed, but severe cases result in lethality in the case of infection or continual relapse and therapy resistance in IBD patients. Thus, understanding the drivers of disease severity will uncover improved strategies for disease management and alternative therapeutic approaches.

A widely used murine model of IBD is chemically induced colitis using dextran sulphate sodium (DSS). DSS is delivered via drinking water and induces tissue damage and inflammation, which most closely resembles human UC. Although the mechanism by which DSS triggers colitis is not well defined, the severity of disease can be modified based on the concentration and duration of exposure.^2^

The natural mouse pathogen *Citrobacter rodentium* has been extensively used to model disease caused by the human gastrointestinal pathogens enteropathogenic and enterohaemorrhagic *Escherichia coli* (EPEC and EHEC) in a true physiological host.^3–5^ Due to the similarities in disease, *C. rodentium* infection can also help elucidate the mechanisms of IBD pathogenesis.^6^ Using a type III secretion system, which injects a repertoire of effector proteins into intestinal epithelial cells (IECs). *Citrobacter rodentium* intimately binds to the colonic epithelium, where it causes intestinal inflammation and tissue damage.^3^ Moreover, shortly after mucosal colonisation *C. rodentium* triggers extensive reprogramming of cell proliferation and metabolic processes in IECs.^7–9^ Importantly, the severity of colitis differs depending on the mouse strain. While some strains (e.g. C57BL/6) infected with *C. rodentium* present self-limiting mild colitis, others (e.g. C3H derivates and FVB) present severe symptoms, including signs of discomfort, diarrhoea and weight loss, which is fatal.^3,10^ Differences in host genetics such as *R-spondin 2* expression and the composition of the gut microbiota have been previously identified as factors, which determine colitis severity upon *C. rodentium* infection.^11–13^

In this study, we tested the hypothesis that uncontrolled neutrophil responses contribute to colitis severity. Neutrophils kill pathogenic microorganisms by phagocytosis, neutrophil extracellular trap formation (NETosis) and/or by releasing the toxic contents of their granules in the extracellular milieu (degranulation). Neutrophils are required for the clearance of *C. rodentium*; however, the mechanisms are largely unknown.^14^ Recently, it has been shown that *C. rodentium* triggers NETosis in vitro and that NET formation is required for clearance in vivo.^15,16^ Both NETosis and degranulation results in the release of reactive oxygen and nitrogen species (ROS and RNS), peroxidases including myeloperoxidase (MPO) and proteolytic enzymes such as neutrophil elastase (NE), cathepsin G and proteinase 3, which are contained within azurophilic granules.^17,18^ These potent agents are unable to distinguish host cells from bacterial targets and if not tightly controlled have damaging consequences on host tissue.

To control the unintentional damaging effects of extracellular proteases, affected tissues secrete specific inhibitors that form stable complexes with proteases and block their activity. For example, NE can be inhibited by Elafin (human-specific), secretory leucocyte protease inhibitor (SLPI) and members of the serpin superfamily, for example, SERPINA1.^19,20^ Serine protease inhibitors (Serpins) are suicide substrates that contain a reactive centre loop (RCL), which is directly targeted by their cognate protease.^21^ Cleavage of the inhibitor distorts the catalytic site of the protease, leading to an irreversible interaction with the Serpin. In mice, SerpinA3N has been reported to be the inhibitor of NE released from T lymphocytes in an in vivo model of neuropathic pain.^22^ SerpinA3N is considered the murine orthologue of human α-1-antichymotrypsin (SERPINA3); however, the RCL of SerpinA3N is identical to α-1-antitrypsin (SERPINA1) and has been reported to share functions of human SERPINA1 and SERPINA3, including the ability to inhibit NE.^23^

Here we compared mild and severe models of infection- and chemical-induced colitis and found that mice exhibiting severe colitis have measurable levels of NE activity in faeces. Mechanistically, we show that this is due to an imbalance in NE and a *se*rine *p*rotease *in*hibitor, *Serpin*A3N, which is produced by IECs during *C. rodentium* infection. By profiling IBD patient samples with active and inactive disease, we reveal that NE activity also reflects colitis severity in humans. Consistent with the notion that NE activity contributes to colitis, disease severity was reduced by infection with an engineered *C. rodentium* strain secreting SerpinA3N. Together, our data illustrate that the balance between antiproteases, such as SerpinA3N, and NE reflects colitis severity.

## Results

### Mice suffering from severe *C. rodentium*-induced disease exhibit extensive intestinal cell death

**Fig. 1:**
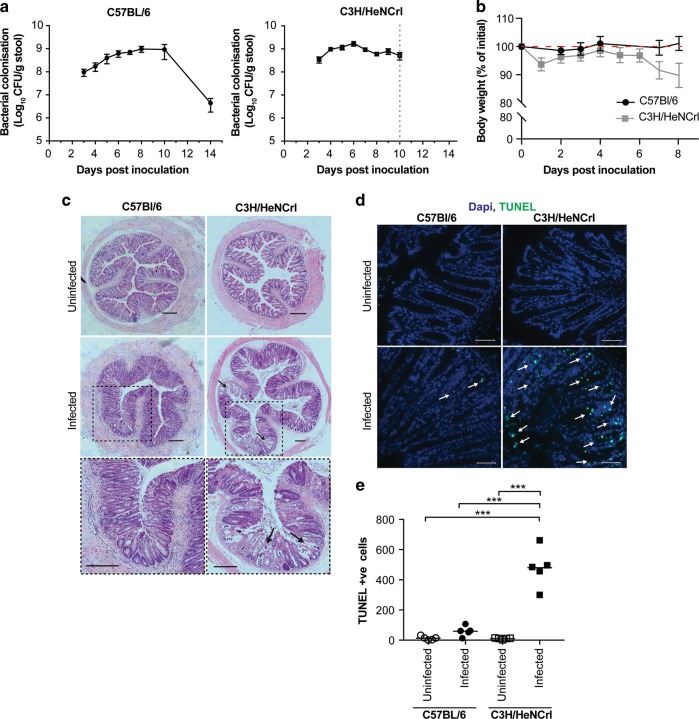
Disease severity induced by intestinal infection differs depending on mouse strain. **a** Intestinal colonisation of *C. rodentium* as measured by CFU/g of stool in C57 and C3H mice. Data are represented as mean ± SD, *n* = 10. **b** Weights presented as percentage of initial weight. C57 mice plotted in black circles and C3H in grey squares. Data are represented as mean ± SD, *n* = 10. **c** Histological analysis of colonic sections; black arrows indicate erosion of epithelium observed only in C3H mice. Scale = 200 μM. **d** Representative images of TUNEL-stained colon sections from infected mice quantified in **e**. Green: TUNEL; blue: DAPI. Scale = 200 μM. **e** Quantification of the number of TUNEL-positive cells per transverse section of colons from individual mice. Multiple comparison one-way ANOVA, *** *P* ≤ 0.001.

To further our understanding of the mechanisms driving colitis, we have focused on the two mouse strains, C57BL/6 (C57) and C3H/HeNCrl (C3H), which display different disease severities.^4,10^ As previously reported, we observed that *C. rodentium* causes a self-limiting infection in C57 mice, which display mild disease pathology with little to no weight loss over the duration of infection (Fig. 1a, b). In contrast, C3H mice succumb to infection as bacterial colonisation peaks earlier and results in high levels of weight loss and diarrhoea (Fig. 1a, b). Colonic crypt hyperplasia (CCH), a key characteristic of *C. rodentium* infection was observed in both mouse strains (Fig. S1)

To assess the extent of tissue damage, we performed hematoxylin and eosin staining on colonic tissue sections collected at the peak of infection, when *C. rodentium* numbers had reached maximal levels of 1 × 10^9^ CFU/g of faeces. This corresponds to 6 days post inoculation (dpi) for C3H and 8 dpi for C57 mice (Fig. 1a). This revealed that mice C3H mice have extensive tissue damage and erosion of the epithelium compared to infected C57 mice (Fig. 1c, arrows). To determine the extent of damage at the cellular level, TUNEL staining was performed to measure the number of apoptotic cells in an infected colon. As expected, uninfected tissue from both mouse strains had very few (<15 TUNEL-positive cells; Fig. 1d, e). Infected C57 mice had an average of 59.4 TUNEL-positive cells per transverse colon section, whereas infected C3H mice had an average of 480 TUNEL-positive cells (Fig. 1e). Accordingly, the severe disease pathology observed in the C3H mice following *C. rodentium* infection correlates with extensive tissue damage and an increase in apoptotic cells.

**Fig. 2:**
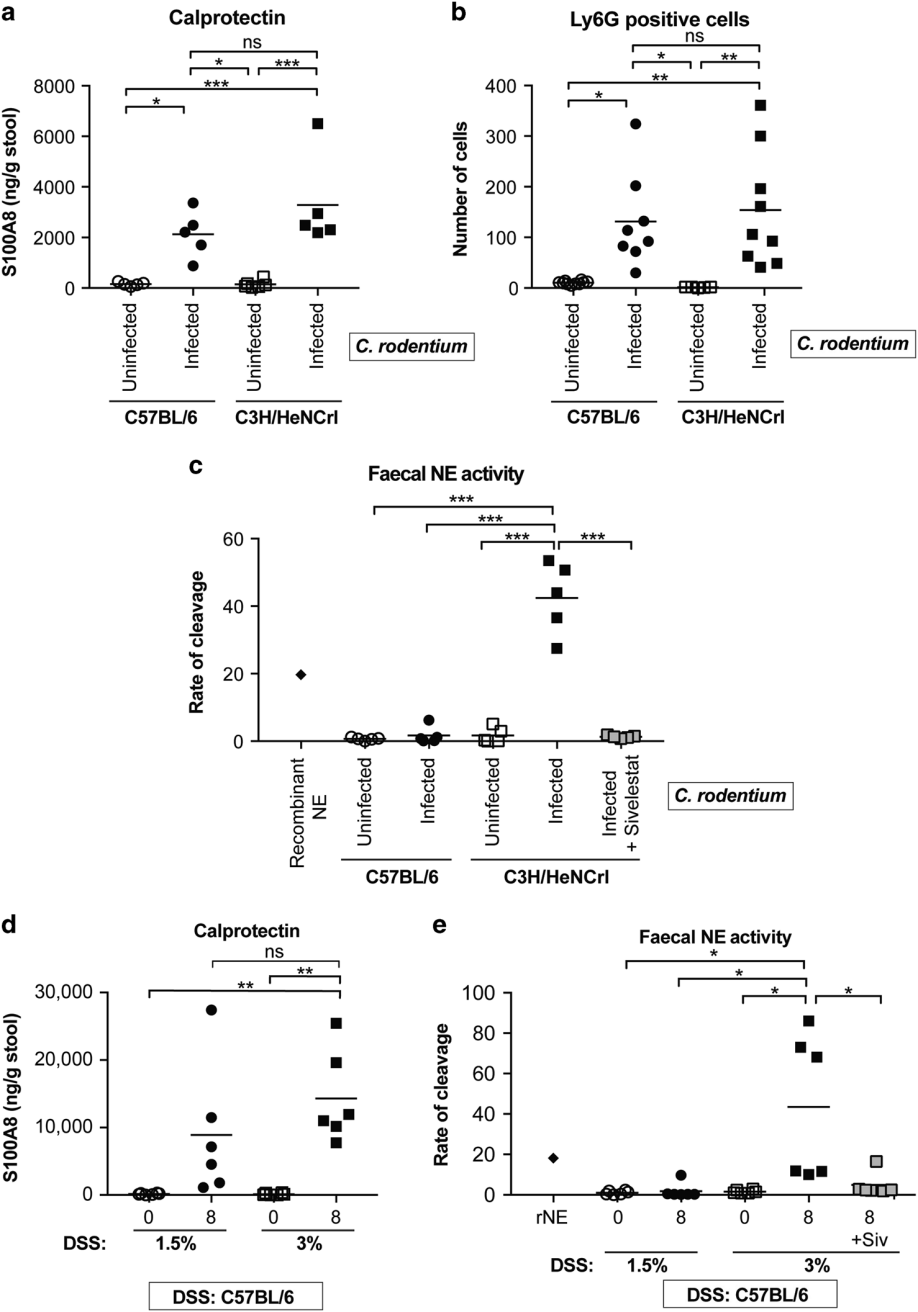
NE activity reflects severity of disease induced by *C. rodentium* and DSS. **a** Levels of S100A8 (calprotectin) in faeces from uninfected and infected mice (8 dpi: C57-closed circles; 6 dpi: C3H-closed squares) as determined by ELISA. Multiple comparison one-way ANOVA, n.s. = non-significant, *P ≤ 0.05, ***P ≤ 0.001. **b** The number of Ly6G-positive cells were counted per colonic section from individual mice (*n* = 8–9). Multiple comparison one-way ANOVA, n.s. = non-significant, *P ≤ 0.05, **P ≤ 0.01. Representative images shown Fig. S2. **c** Measurements of faecal NE activity  in uninfected and infected (C57, 8 dpi-closed circles; C3H, 6 dpi-closed squares) mice, measured using a fluorogenic tetrapeptide substrate. Purified recombinant NE was used as a control (diamond). Grey squares indicate samples treated with Sivelestat. Multiple comparison one-way ANOVA, ***P ≤ 0.001. **d** Faecal S100A8 ELISA (calprotectin) of samples taken from C57 mice pre-treatment (0) and at 8 days post treatment (1.5% DSS- closed circles; 3% DSS- closed squares). Multiple comparison one-way ANOVA, n.s. = non-significant, **P ≤ 0.01. **e** Faecal NE activity in samples from C57 mice pre-treatment (0) and at 8 days post treatment (1.5% DSS- closed circles; 3% DSS- closed squares). Purified rNE was used as a control (diamond). Grey squares indicate samples treated with Sivelestat. Multiple comparison one-way ANOVA, *P ≤ 0.05.

Neutrophil infiltration into the intestinal mucosa is a characteristic of colitis. For example, S100A8 and S100A9 (calprotectin) constitute ~45% of the cytoplasmic content of neutrophils and is used as a non-invasive faecal biomarker for assessing colonic inflammation in IBD.^24^ We therefore tested if neutrophil numbers vary following *C. rodentium* infection of C57 and C3H mice. We measured calprotectin levels (S100A8) by enzyme-linked immunosorbent assay (ELISA) in faecal samples collected from uninfected and infected mice. While calprotectin increased upon infection, no difference was observed between C57 and C3H mice (Fig. 2a). To quantify the number of neutrophils specifically recruited to the colonic mucosa of infected mice, we stained transverse tissue sections with Ly6G, a marker expressed predominantly on neutrophils. In agreement with calprotectin levels, the number of neutrophils infiltrating the mucosa did not differ between mouse strains (Fig. 2b and Fig. S2A). Tissue sections were also stained with an antibody against NE, which is localised within the azurophil granules of neutrophils. NE stained in a granular pattern localised within Ly6G-positive cells, with no apparent difference in intracellular NE staining between C57 and C3H mice (Fig. S2A, B). Together, this indicates that the number of neutrophils at the peak on infection does not differ in mice with mild and severe disease.

### Faecal NE activity is only detected in mice with severe disease

As NE has long been known to be the primary cause of tissue damage in chronic inflammatory conditions of the lung,^25^ we hypothesised that excessive NE release may be contributing to disease severity in *C. rodentium*-infected C3H mice. To test this hypothesis, we first used a commercially available fluorogenic tetrapeptide substrate targeted by NE (Ala-Ala-Pro-Val) to measure faecal NE activity (Fig. S3A). As a positive control, we included recombinant mouse NE (rNE), which efficiently cleaved the substrate (Fig. S3A and Fig. 2c). When the substrate was added to faecal samples, NE activity was only detected in faeces from infected C3H mice and not in samples from uninfected mice or infected C57 mice. Since more specific and sensitive substrates for human NE containing unnatural amino acids have been reported,^26^ we generated one such substrate with a Arg(NO_2_) residue at position P4 amino acid, (Arg(NO_2_)-Met(O2)-Oic-Abu; Fig. S3B). Arg(NO_2_) was chosen because it is a bulky hydrophilic amino acid, which is preferred for both human and mouse NE.^26,27^ Using rNE we demonstrated that the optimised substrate was 58 times better hydrolysed by rNE than the commercial substrate and was therefore used for all subsequent NE activity assays (Fig. S3C). Repeating the assay using the optimised substrate in the mouse faecal samples revealed similar results to the commercial substrate, suggesting that both substrates are indeed being cleaved by NE present in faecal samples from mice with severe disease (Fig. 2c). Furthermore, the addition of NE inhibitor Sivelestat significantly reduced faecal NE activity in C3H mice (Fig. 2c, grey boxes). These data suggest that active NE is present in faeces of mice suffering from severe *C. rodentium* infection, which may be the underlying reason for tissue damage at the inflamed site.

### Faecal NE activity defines disease severity in chemically induced colitis

To determine the role of NE activity in an alternative model of colitis, we utilised the DSS colitis model. To model mild and severe diseases, we used a low (1.5%) and high (3%) dose of DSS in C57 mice, respectively. The difference in disease severity was validated by histological analysis, TUNEL staining, weight loss and shortening of colon length (Fig. S4A–C). Mice treated with 3% DSS reached humane endpoint (20% weight loss) by day 8 whereas mice treated with 1.5% DSS only lost ~5% body mass (Fig. S4C). In addition, histology and TUNEL staining revealed more tissue damage and enhanced cell death in mice with severe disease (3% DSS). Disease activity index (DAI; combination weight loss, diarrhoeal and haematochezia score) was higher in mice with severe disease (3% DSS) compared to mild disease (1.5% DSS; Fig. S4D). S100A8 and MPO ELISA was performed on faecal samples collected prior to treatment and at day eight as surrogate readouts for neutrophil recruitment and release of azurophil granules, respectively. Although increased calprotectin and MPO was observed in faeces from both groups, there was no difference in levels between mice with mild and severe disease, suggesting similar neutrophil recruitment and degranulation at 8 days post treatment (Fig. 2d and Fig. S4E). As we observed for infection-induced colitis, faecal NE activity was only detected in mice with severe colitis (Fig. 2e). Taken together, the correlation between tissue damage and NE activity reflects disease severity in murine models of chemically induced and infection-induced colitis.

### NE activity reflects clinical activity in human IBD

**Fig. 3:**
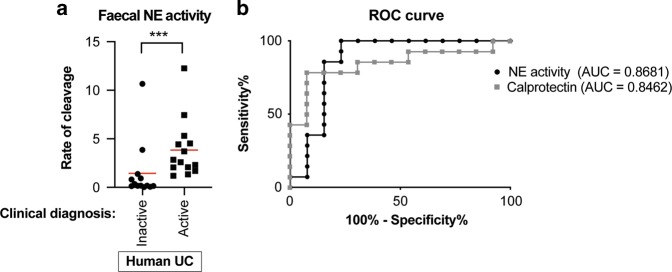
Faecal NE activity predicts clinical activity in UC patients. **a** Mann–Whitney test of rate of cleavage of NE substrate in IBD samples from ulcerative colitis (UC) patients classified as having inactive (circles, *n* = 13) and active (squares, *n* = 14) disease, ***P ≤ 0.001. **b** Receiver operating characteristic (ROC) curve analysis of accuracy of NE activity and faecal calprotectin to predict active and inactive disease. Area under curve (AUC) for NE activity = 0.8681 and 0.8462 for calprotectin.

**Fig. 4:**
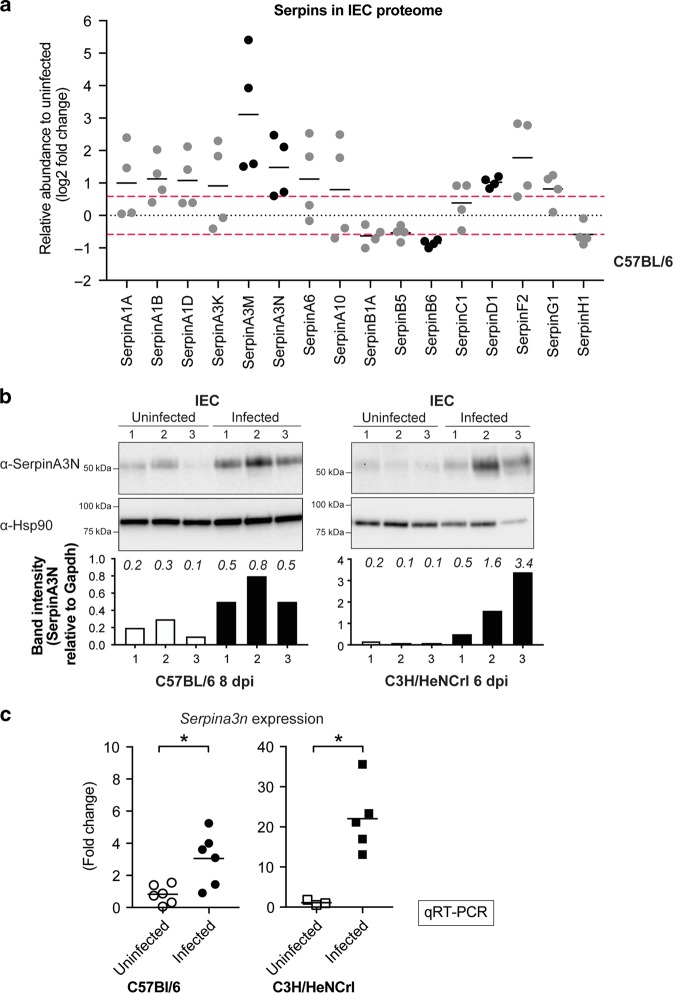
SerpinA3N is produced by infected IECs. **a** Log 2 fold change of Serpins identified in proteomic analysis of IECs from uninfected and infected C57 (8 dpi) mice. Black dots indicate proteins with increased or decreased abundance above or below 1.5-fold change, log 2: 0.585 (red line) in four individual experiments: SerpinA3M, SerpinA3N, SerpinB6, SerpinD1. **b** Western blot analysis using anti-SerpinA3N antibody on enriched IECs samples from three uninfected and three infected C57 and C3H mice. Anti-Hsp90 was used as a loading control. Bar graph below blot is quantification of SerpinA3N band relative to loading control; the actual values are indicated in italics above each bar. **c** mRNA levels of *Serpina3n* expressed by infected and uninfected IECs measured by qRT-PCR relative to *Gapdh* control. Values are expressed as ΔΔCT relative to uninfected, Mann–Whitney test, *P ≤ 0.05.

To translate our findings to human disease, we measured faecal NE activity in samples from patients with active and inactive UC and compared it to their clinical DAI (Table 1). Based on previous studies, a DAI of ≥5 was used as a sign of clinical relapse and therefore active disease.^28^ Samples were also sent for calprotectin measurements to enable a direct comparison with the current most commonly used faecal marker. The NE activity test was performed by measuring the rate of substrate cleavage for each sample and the rate grouped based on clinical activity: a significant difference was observed between patients classified as having inactive or active disease (Fig. 3a). To calculate the diagnostic ability of NE activity a receiver operator characteristic (ROC) curve analysis was performed: the areas under curve (AUC) obtained were 0.8681 and 0.8462 for faecal NE activity and calprotectin measurements, respectively (Fig. 4b). While the sample size is small (*n* = 27), these analyses have directly compared NE activity with calprotectin levels in the same faecal sample. Furthermore, the AUC obtained for calprotectin is similar to the results of previous studies.^29^ This suggests that faecal NE activity predicts clinical DAI with comparable accuracy to calprotectin and therefore may offer an additional faecal marker for identifying disease activity in UC patients.

**Table 1:** Clinical characteristics of human UC patients

Distribution *N* (%)	
Proctitis	6 (22%)
Left sided UC	9 (33%)
Pancolitis	12 (45%)
Gender *N* (%)	
Male	11 (41%)
Female	16 (59%)
Mean age of patients	39.9 (range 18–72)
Activity (SCCAI)	
<5 (inactive)	13
≥5 (active)	14

### Inflamed IECs secrete SerpinA3N

To protect against extracellular proteases activity inflamed mucosa secrete inhibitors that form stable complexes with the proteases. We therefore hypothesised that the observed faecal NE activity in C57 and C3H mice reflects differential expression of cognate Serpins by IECs, as a means for protection against NE-induced damage. We took advantage of the global proteomic analysis we had previously performed on IECs enriched from colons of infected C57 mice.^7,30^ By comparing infected to uninfected IECs, an increased abundance of >1.5-fold change (log 2 >0.58) was observed for SerpinD1, SerpinA3M and SerpinA3N in four biological repeats. (Fig. 4a). Importantly, SerpinA3M and SerpinA3N are members of the same Serpin family (α-1-antichymotrypsin) and SerpinA3N is a previously reported inhibitor of NE in vivo.^22^ Western blot analysis of SerpinA3N in IECs from C57 and C3H mice revealed an increased abundance in both strains upon infection (Fig. 4b). Next, to determine whether *Serpina3n* is transcriptionally induced in response to infection, we performed quantitative real-time PCR (qRT-PCR). *Serpina3n* expression was increased at the peak of infection by ~3-fold and 20-fold in C57 and C3H mice, respectively (Fig. 4c). Of note, *Serpina3m* was undetectable in both uninfected and infected IECs from both mouse strains. These results indicate that SerpinA3N is transcriptionally induced and expressed by IECs following *C. rodentium* infection. As SerpinA3N is produced by IECs in both C57 and C3H mice, we hypothesised that the level of SerpinA3N in C3H mice is insufficient to neutralise luminal NE activity.

### Proteolytic activity of NE is balanced by SerpinA3N in mild disease

**Fig. 5:**
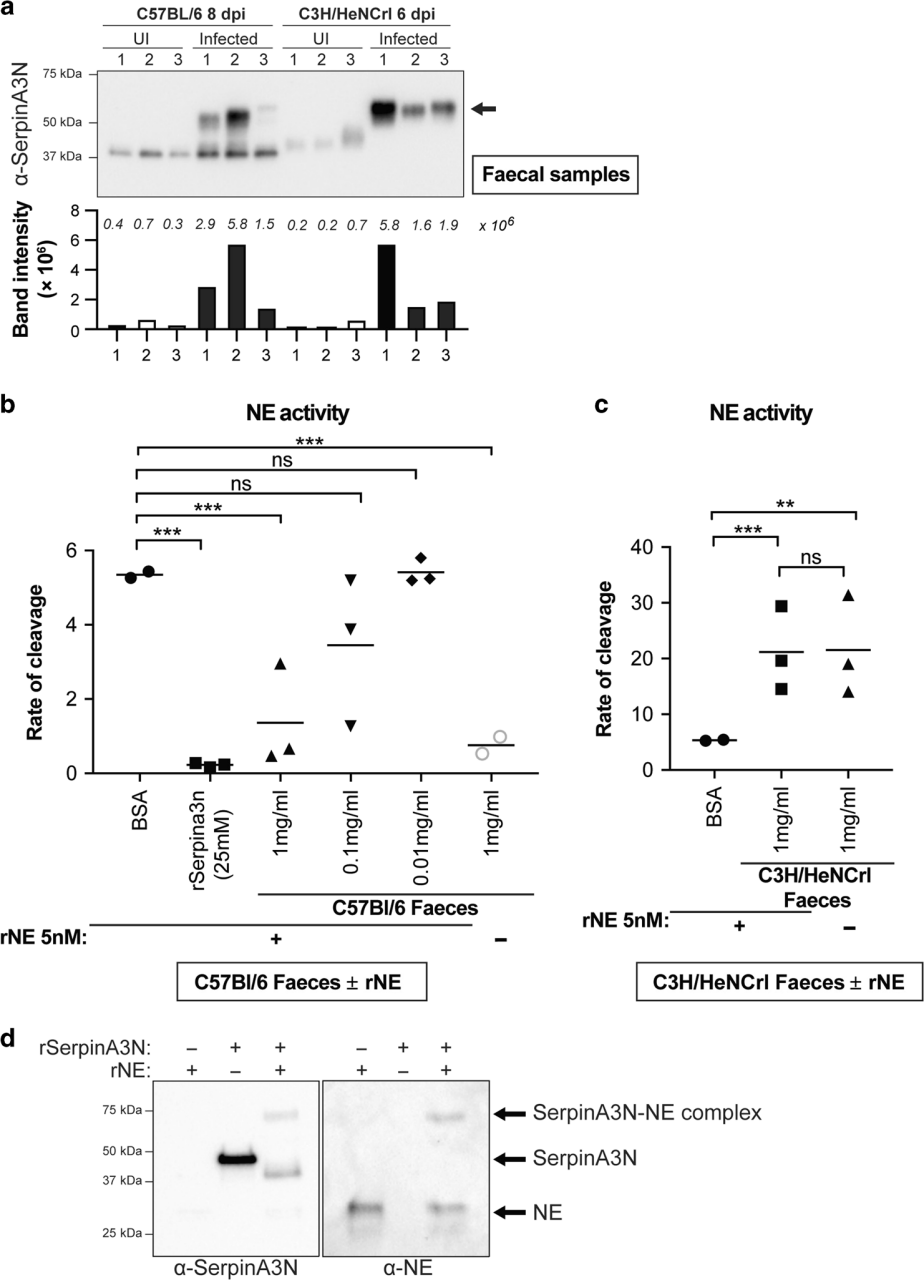
SerpinA3N is secreted during infection and inhibits NE activity. **a** Western blot analysis of faecal samples taken from three uninfected and three infected C57 (8 dpi) and C3H (6 dpi) mice using anti-SerpinA3N antibody. Free SerpinA3N (ca. 40 kDa) is detected in uninfected mice and infected C57. In both C57 and C3H mice, a band at ~55 kDa (arrow) corresponds to a protease–SerpinA3N complex. Bar graph below blot is quantification band/s intensity; actual values are indicated in italics above each bar. **b** Faecal samples from infected C57 mice (1 mg/ml- triangles) significantly inhibit recombinant NE (rNE) activity whereas BSA (circles) or lower concentrations (0.1mg/ml-inverted triangles; 0.01mg/ml-diamonds) did not. Recombinant SerpinA3N inhibits rNE (squares). Grey circles are NE activity in faecal samples from C57 mice without rNE. Multiple comparison one-way ANOVA, n.s. = non-significant, ***P ≤ 0.001. **c** Rate of NE substrate cleavage by rNE pre-incubated with faecal samples from C3H (squares) or BSA (circles). Multiple comparison one-way ANOVA, n.s. = non-significant, **P ≤ 0.01, ***P ≤ 0.001. **d** Western blot analysis of recombinant (r) SerpinA3N incubated with or without activated rNE. Band at ca. 75 kDa is complexed SerpinA3N and NE, which is present in both SerpinA3N and NE blots.

Although produced by IECs, SerpinA3N is secreted to function extracellularly. Therefore, to analyse the presence of extracellular SerpinA3N, we performed western blot analysis of faecal samples collected from C57 and C3H mice at the peak of *C. rodentium* colonisation. Immunoblotting of faecal samples from uninfected mice, using a SerpinA3N antibody, identified a single faint band at ~40 kDa (Fig. 5a). In contrast, two main products were detected in faecal samples from infected C57 mice (mild disease). The lower band corresponds to free SerpinA3N, while the band at 65 kDa is SerpinA3N in a complex with a protease, since the irreversible protease–serpin interaction is not disrupted by denaturation. The difference in molecular weight between the free SerpinA3N and the protease–SerpinA3N complex of ~25 kDa corresponds to the molecular weight of NE. We also probed for another reported murine NE inhibitor, SLPI. Although detected in infected IECs, no SLPI was present in faeces, suggesting that it is not secreted or unstable and that SerpinA3N is the main inhibitor of NE in an inflamed colon (Fig. S5). Importantly, while the SerpinA3N–protease complex was detected in faecal samples from infected C3H mice, the free SerpinA3N was undetectable (Fig. 5a). Based on these observations, we hypothesised that the balance between SerpinA3N and NE in mild disease (C57) is in favour of the inhibitor (i.e. excess free SerpinA3N), whereas in mice with severe disease (C3H), the balance is in favour of NE (i.e. insufficient SerpinA3N to neutralise luminal NE).

To test the hypothesis that mice with mild disease have surplus SerpinA3N, we incubated active recombinant NE with faecal samples from infected C57 mice. As positive control, we demonstrated that recombinant (r) SerpinA3N efficiently inhibited rNE activity (Fig. 5b and Fig. S3D, E). Furthermore, western blot analysis of rSerpinA3N incubated with rNE revealed a SerpinA3N-NE complex at 75 kDa using both SerpinA3N and NE antibodies (Fig. 5d). Of note, rSerpinA3N runs at a higher molecular weight than faecal SerpinA3N, likely due to cleavage of SerpinA3N by unknown protease/s present in feaces. Next, we added increasing amounts of C57 faecal sample (excess SerpinA3N) to activated rNE; 1 mg/ml of faeces significantly inhibited rNE activity (Fig. 5b). As a control, incubation of rNE with faecal samples from infected C3H, which have active NE, did not alter NE activity (Fig. 5c). Together, this supports the notion that faecal samples from infected C57 mice have SerpinA3N in excess, whereas C3H mice have excessive NE.

### Neutrophils in mice with severe colitis degranulate earlier than mice with mild disease

**Fig. 6:**
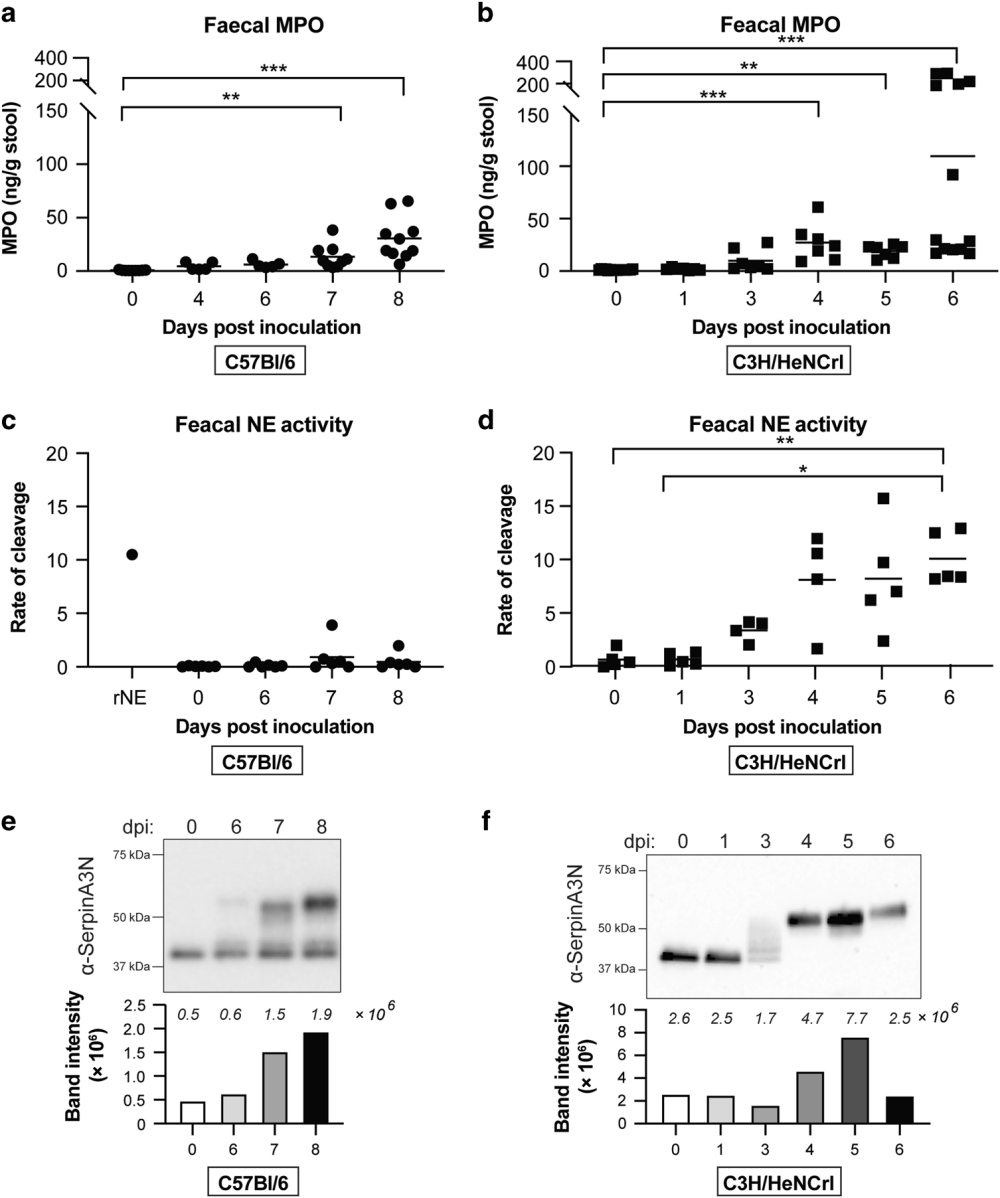
Neutrophils in C3H mice degranulate earlier than C57 mice. Levels of MPO in faeces from infected C57 (**a**) or C3H (**b**) mice over time as determined by ELISA. Multiple comparison one-way ANOVA, **P ≤ 0.01, ***P ≤ 0.001. **c** Analysis of faecal NE activity in infected C57 mice collected overtime. Multiple comparison one-way ANOVA was performed, no significant difference. **d** Analysis of faecal NE activity in infected C3H mice collected overtime. Multiple comparison one-way ANOVA was performed, *P ≤ 0.05, **P ≤ 0.01. **e** Western blot analysis using anti-SerpinA3N antibody of faecal samples taken from infected C57 (**e**) or C3H (**f**) mice over time. Lower band (ca. 40 kDa) is free SerpinA3N and upper band (>50 kDa) is SerpinA3N in complex with protease. Bar graphs below each blot is quantification band/s intensity; the actual values are indicated in italics above each bar.

To investigate why NE-SerpinA3N is imbalanced in C3H mice, faecal samples were collected at timepoints leading up to the peak of infection. Calprotectin levels are significantly increased at 4 dpi in both C57 and C3H mice (Fig. S6).^8^ This suggests similar temporal neutrophil recruitment irrespective of mouse strain. We therefore hypothesised that the differences between the two mouse strains is due degranulation. To test this, we quantified MPO levels in faeces by ELISA. Released MPO levels were significantly higher 3 days earlier in C3H mice compared to C57 (day 4 vs. day 7; Fig. 6a, b). As MPO is present in azurophilic granules, like NE, this suggests that NE is also released at this early timepoint in C3H mice. We therefore measured faecal NE activity and performed western analysis of SerpinA3N over time. In C3H mice, faecal NE activity was detected from 3 dpi, which corresponds to smearing pattern of SerpinA3N by western blot (Fig. 6d, f). From 4 dpi all SerpinA3N is in a higher molecular weight complex, correlating with high NE activity. In contrast, no faecal NE activity was detected in infected C57 mice and free SerpinA3N was present at all timepoints (Fig. 6c, e). Together, these results suggest that neutrophils in C3H mice undergo degranulation much earlier than C57 mice, leading to an accumulation of NE activity which saturates all available SerpinA3N.

### *Citrobacter rodentium* producing SerpinA3N protects mice from tissue damage

**Fig. 7:**
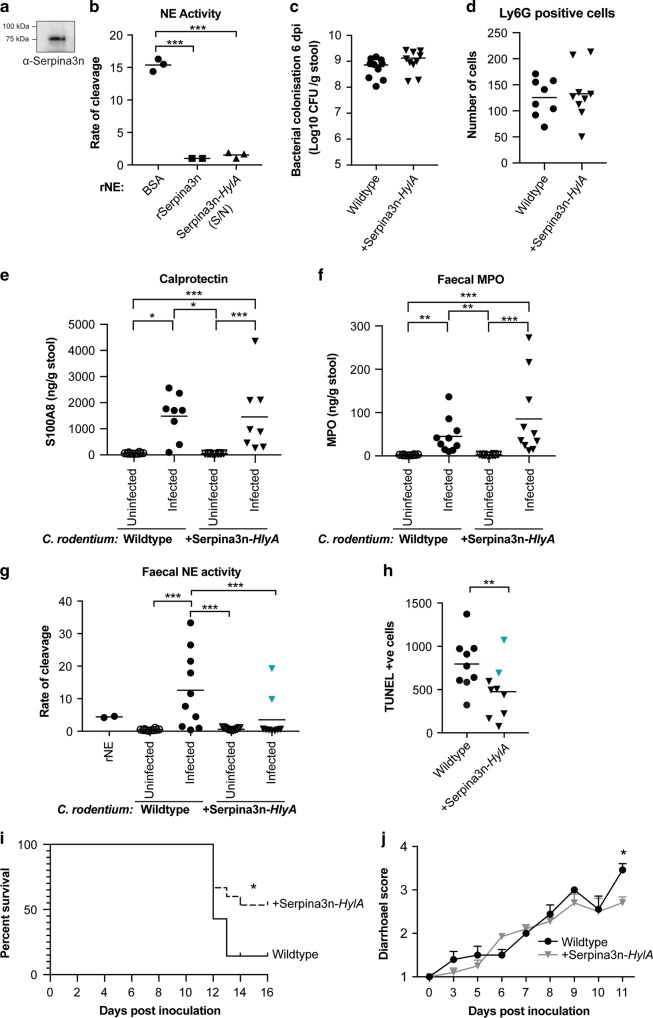
Delivery of SerpinA3N at the site of infection reduces faecal NE activity and disease severity in C3H mice. **a** Western blot analysis of SerpinA3N-HylA in a culture supernatant obtained from ICC2031. **b** Rate of cleavage of NE substrate by 10 nM recombinant neutrophil elastase (rNE) following incubation with BSA (control), affinity-purified SerpinA3N-HlyA and rSerpinA3N. **c** Shedding of WT *C. rodentium* (circles) and ICC2031 (inverted triangles) upon infection of C3H mice. Number of CFU/g of stool was measured 6 dpi. **d** The number of Ly6G-positive cells were counted per colonic section from individual mice (*n* = 8–9). **e** S100A8 ELISA (calprotectin) shows no significant difference between mice infected with WT *C. rodentium* (circles) and ICC2031 (inverted triangles)  at 6 dpi. Multiple comparison one-way ANOVA was performed; *P ≤ 0.05, ***P ≤ 0.001. **f** MPO ELISA shows no significant difference between mice infected with WT *C. rodentium* (circles) and ICC2031 (inverted triangles) at 6 dpi. Multiple comparison one-way ANOVA was performed; **P ≤ 0.01, ***P ≤ 0.001. **g** Analysis of faecal NE activity in samples collected pre-infection (uninfected) and 6 dpi. Cyan inverted triangles indicates outliers determined by ROUT test, *Q* = 0.5%. Multiple comparison one-way ANOVA was performed, ***P ≤ 0.001. **h** Quantification of the number of TUNEL-positive cells per transverse section of colons from individual mice. Cyan inverted triangles correspond to outliers based on NE activity. Mann–Whitney test, **P ≤ 0.01. **i** Survival curve of C3H mice infected with WT *C. rodentium* (*n* = 14) and ICC2031 (*n* = 15); combined data from three biological repeats. Comparison of survival curves by log-rank (Mantel–Cox) test; *P = 0.0302. **j** Diarrhoeal score of mice infected with WT *C. rodentium* (*n* = 14; black line) and ICC2031 (*n* = 15; grey line) over time; combined data from three biological repeats. Multiple *t* tests, *P ≤ 0.05 (11 dpi).

To test whether we could tilt the balance in favour of SerpinA3N in C3H mice, we engineered a *C. rodentium* strain that produces and secretes mouse SerpinA3N into the extracellular milieu. We fused a His-tagged SerpinA3N lacking the eukaryotic signal peptide to the bacterial HlyA secretion signal for expression in *C. rodentium*. The type I/HlyA secretion system allows the secretion of proteins directly from the bacterial cytoplasm.^31^ The supernatant obtained after growing this strain was affinity purified and SerpinA3N-HlyA was detected by western blotting (Fig. 7a). To measure the inhibitory capacity of secreted His-SerpinA3N-HlyA, rNE was incubated with the purified protein and NE activity measured. SerpinA3N-HylA inhibited rNE as efficiently as recombinantly expressed His-SerpinA3N (Fig. 7b and Fig. S3D, E). Once the inhibitory activity of the SerpinA3N-HlyA was confirmed, we engineered a *C. rodentium* strain (named ICC2031) that constrictively expresses the SerpinA3N-HlyA operon from the chromosome. ICC2031 had no growth defect when grown in vitro in minimal medium; moreover, ICC2031 colonisation in vivo and CCH was comparable to the parental wild-type (WT) strain (Fig. S7A, B and Fig. 7c). Furthermore, neutrophil recruitment and release of azurophil granules was similar between mice infected with the WT and ICC2031 strains at peak of infection (Fig. 7d–f). However, NE activity at the peak of infection was significantly reduced in C3H mice infected with the ICC2031 compared to WT *C. rodentium*, indicating that the reduction in NE activity is specifically due to the secretion of SerpinA3N (Fig. 7g).

**Fig. 8:**
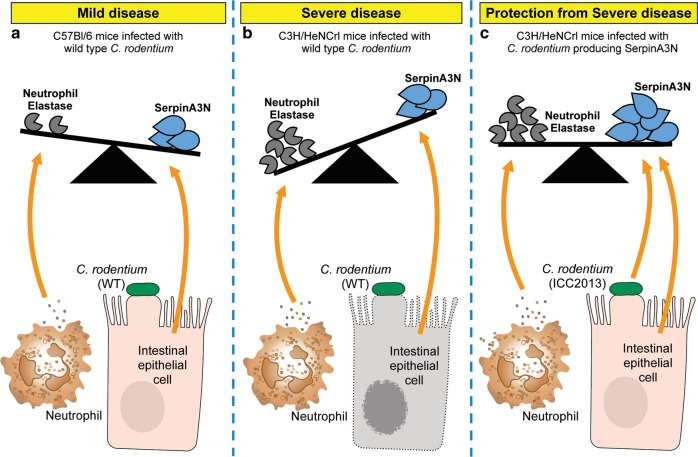
Model demonstrating the interplay between NE and SerpinA3N during infection. **a** *Citrobacter rodentium* infection induces the recruitment of neutrophils. In response to inflammatory triggers NE is released. SerpinA3N is produced and secreted by infected IECs to protect from NE-induced damage. In mild disease (e.g. C57 mice), the balance is in favour of the inhibitor (i.e. excess free SerpinA3N). **b** In severe disease (e.g. C3H mice), there is insufficient SerpinA3N to neutralise excess luminal NE activity, which results in IEC cell death (grey cell with fragmented membrane and nuclei). **c** The balance can be pushed in favour of the inhibitor in C3H mice using engineered *C. rodentium* to deliver SerpinA3N.

Based on our working hypothesis that excessive NE activity is contributing to disease severity in C3H mice, the reduction in NE activity after infection with ICC2031 should result in decreased tissue damage. To determine the extent of damage, we collected colons from infected C3H mice at 6 dpi and performed TUNEL staining. Compared to WT infection, mice infected with ICC2031 have significantly reduced TUNEL-positive cells (Fig. 7h). Of note, the extent of tissue damage correlated with the amount of NE activity in the corresponding faecal sample because outlier samples based on the NE activity assay also had the opposite results in the TUNEL analysis (Fig. 7g, h, highlighted points). To determine the contribution of NE to disease severity, we infected mice with WT and ICC2013 and continued the experiment until mice reached humane endpoint (20% weight loss). Only 14% of mice infected with WT *C. rodentium* survived infection, whereas 53% of infected with ICC2031 survived (Fig. 7i). In addition, mice infected with ICC2031 had a reduced diarrhoeal score at 11 dpi (Fig. 7j). We also observed a similar trend treating mice intrarectally with Sivelestat (Fig. S8A–D). These results corroborate the notion that in a hyper-inflamed colon, high NE levels overwhelms the available SerpinA3N resulting in tissue damage, which contributes to disease severity (Fig. 8).

## Discussion

*Citrobacter rodentium* has been widely used to model human enteric pathogens in the context of a physiological host. However, the inflammatory signatures of *C. rodentium* infection closely resemble those seen in IBD, including neutrophil infiltration, mucosal damage and the involvement of key cytokines and innate immune receptors, such as NOD2.^3,32^ Therefore, *C. rodentium* infection is emerging as a model of IBD. Here, we show that NE is released and drives mucosal tissue damage during infectious colitis. By extrapolating from our findings using *C. rodentium*, we reveal that faecal activity of NE also reflects disease severity in the DSS colitis model as well as in human IBD. Our proof-of-concept study using faeces from patients with UC suggests that faecal NE activity is equivalent to the current gold-standard maker calprotectin for predicting active disease in UC patients. However, in the murine models of infection-induced and chemically induced colitis, NE activity successfully distinguished between disease severity, whereas calprotectin did not. Calprotectin is released from neutrophils early during inflammation and does not directly contribute to tissue damage.^33^ In contrast, NE is released as a last line of defence usually from damaged or dying neutrophils and active NE directly contributes to tissue damage at inflamed sites.^34^ Therefore, faecal NE activity is an ideal additional marker for determining clinical activity in UC and should be investigated in a larger patient cohort.

Under baseline conditions, the NE is controlled by intracellular compartmentalisation within granules of neutrophils, and if released, its activity is antagonised by extracellular inhibitors. The continual and excessive release of NE at inflammatory sites or disruption to inhibitor function can push the balance in favour of NE activity, which causes tissue injury. Elevated levels of NE are detected in colonic mucosal tissue and plasma samples from UC patients and therefore measurement of NE abundance has previously been explored as a biomarker for IBD.^35–37^ However, NE abundance does reliably indicate active disease in IBD,^38^ and is inferior to other faecal markers, for example, calprotectin and lactoferrin.^35^ This is likely due to the concatenate release of endogenous NE inhibitors. Corroborating this notion, the human orthologues of SerpinA3N, α-1-antitrypsin (SERPINA1) and α-1-antichymotrypsin (SERPINA3) are increased in serum and faeces from IBD patients.^39–41^ These previous studies have focused on total protein levels and not the actual activity of NE. In this study, using the *C. rodentium* infection model, we demonstrate that SerpinA3N is produced and released by infected IECs to mitigate NE activity. To our knowledge, this is the first time a host Serpin has been implicated in infectious colitis. In mice with mild disease symptoms, no faecal NE activity is detected due to sufficient expression of SerpinA3N. In contrast, in mice suffering from severe symptoms, faecal NE activity results from an imbalance with SerpinA3N, likely due to the prolonged neutrophil recruitment and/or excessive release of NE (Fig. 8).

Due to the damaging effects of uncontrolled NE activity, NE has been explored as a potential therapeutic target using the DSS colitis model. Previous studies have demonstrated that treatment with Sivelestat or administration of SERPINA1 and Elafin (human NE inhibitors) attenuates disease.^42–44^ In contrast to these studies, we have used a natural murine NE inhibitor, SerpinA3N, to target NE in a physiological model of infection-induced colitis. By using the pathogen to specifically deliver SerpinA3N to the site of infection, we observed reduced NE activity and tissue damage. This suggests that delivering Serpins specifically to the inflamed site, that is, using engineered commensal Gram-negative bacteria, may be useful for treating inflammatory diarrheal diseases. In addition, there is an urgent need for alternative therapies for IBD patients, which do not respond to current first-line treatments.^45^ To help meet this clinical gap, we propose the possibility of screening specifically for patients for NE activity and treating this subset with NE inhibitors.

In summary, our results demonstrate that uncontrolled neutrophil responses contribute to the detriment of the host. During colitis induced by pathogen or as a result of chronic disease extracellular NE is regulated by antiproteases such as SerpinA3N. However, an imbalance resulting in excessive NE activity causes tissue damage, which likely heightens colitis pathology (Fig. 8). Thus, our results reveal that it is NE activity not NE abundance that should be the focus of prognostic tests for IBD management. Furthermore, modifying the NE-antiprotease balance by employing bacteria to deliver auxiliary Serpins specifically at the inflammatory site offers a novel approach for treating enteric infections and IBD.

## Materials and methods

### Patient sample collection

Faecal samples were collected from patients with confirmed IBD, diagnosed by experienced gastroenterologists. Samples collected within 6 h of production were divided and stored at −80 °C or sent for calprotectin analysis. Clinical data, including disease type and location, were recorded and each patient’s DAI calculated (Table 1). The DAI was determined using the simple clinical colitis activity index (SCCAI). Ethical approval for the research was given by Stanmore REC (IRAS 243310).

### In vivo infection

Animal experiments complied with the Animals Scientific Procedures Act 1986 and UK Home Office guidelines and were approved by the local ethical review committee. Experiments were designed in agreement with the ARRIVE guidelines^46^ for the reporting and execution of animal experiments, including sample randomisation and blinding. Mouse experiments were performed with 3–5 mice/group and repeated at least two times independently. Pathogen-free female 18–20 g C57 and 20–25 g C3H mice (Charles River Laboratories, Italy) were housed in high-efficiency particulate air-filtered cages with sterile bedding and given ad libitum access to food and water. Mice were infected with *C. rodentium* by oral gavage and bacterial colonisation monitored.

### DSS treatment

DSS was added to sterilised water at a concentration of 1.5% or 3% (wt/vol) and administered to C57 mice for 6 days *ad libitum*. At day 6, DSS was replaced with sterilised water and mice were harvested 2 days later. Mice were weighed and faecal samples collected over the duration of the experiment and DIA recorded (Table S3).

### Faecal sample collection and protein extraction

Faecal samples were collected in LoBind tubes (Eppendorf), placed on ice and resuspended at 1 g/10 ml in cold faecal protein extraction buffer (FPEB; 50 mM Tris, pH 7.5, 150 mM NaCl). Samples were homogenised and incubated on ice for 30 min with a brief vortex every 5 min. Samples were centrifuged at 2000 × *g* at 4 °C for 10 min. The supernatant (S/N) was collected and filtered using a 0.2 μM column (Costar) by centrifugation at 15,800 × *g* at 4 °C for 30 min. The flow through was transferred to a LoBind tube and stored at −80 °C. Human faecal samples were defrosted on ice and processed as described.

### NE activity assay

NE activity was measured in faecal samples or recombinant (r) protein pre-mixes through the fluorescence released after NE-induced cleavage of MeOSuc-Ala-Ala-Pro-Val-AMC (Bachem) at a final concentration of 100 μM or MeOSuc-Arg(NO_2_)-Met(O2)-Oic-Abu-ACC at a final concentration of 10 μM. For MeOSuc-Ala-Ala-Pro-Val-AMC, 25 μl of mouse faecal sample was used. For MeOSuc-Arg(NO_2_)-Met(O2)-Oic-Abu-ACC, mouse or human faecal samples were diluted 1:5 or 1:25 in FPEB (±10 μM Sivelestat), respectively. Samples were mixed 1:1 with the NE substrate diluted in NE assay buffer to a final volume of 50 μl. Fluorescence was measured using FLUOstar Omega microplate reader (BMG Labtech) at excitation/emission wavelengths of 355 nm/460 nm up to 2 h, with a 30 s interval time. A linear regression fit on the linear portion of each progress curve was performed to calculate the slope of line (rate of substrate cleavage). A multiple comparison one-way ANOVA (analysis of variance) was performed comparing the slope of the regression lines (Prism 7).

Mouse rNE was activated by incubating with cathespin C for 2 h at 37 °C in 50 mM MES (2-(*N*-morpholino)ethanesulfonic acid), pH 5.5, and diluted to 1–5 nM in NE assay buffer in a 20 μl volume and mixed with rSerpinA3N (3.125–100 nM), faecal sample, purified culture supernatant (SerpinA3N-HylA), Sivelestat or bovine serum albumin (BSA) diluted in FPEB to 20 μl volume. Pre-mixes were incubated in a black-walled 96-well plate for 30 min at 37 °C prior to the addition of the optimised NE substrate diluted in NE assay buffer to 10 μl. Readings were measured immediately, and analysis performed as described above.

### Western analysis

Enterocytes were lysed in Triton lysis buffer + 1% sodium dodecyl sulfate (SDS), passed through 0.8 ml columns (Pierce) to shred genomic DNA and protein concentration was estimated using the BCA Protein Assay Kit (Pierce). Faecal extracts were lysed directly in 6× SDS loading dye. Proteins were separated by SDS-polyacrylamide gel electrophoresis using 4–20% TGX gels (Bio-Rad) and transferred to PVDF membranes using the Bio-Rad Trans-Blot^®^ semi-dry transfer cell. Membranes were blocked for 1 h with 5% skim milk/TBST before sequential incubation with primary antibodies in 5% BSA/TBST and secondary antibodies in 5% skim milk/TBST for 1 h each with washing in between. Blots were visualised using Clarity ECL (Bio-Rad) and the ChemiDoc^TM^ imaging system (Bio-Rad). Bands were quantified using the Image Lab software (Bio-Rad).

## Supplementary information

Supplementary Materials
