# Supplementary material for: Faecal neutrophil elastase-antiprotease balance reflects colitis severity
Source: Mucosal Immunol. 2019 Nov 26;13(2):322–33. doi: 10.1038/s41385-019-0235-4 (PMC7039808; doi:10.1038/s41385-019-0235-4)
Supplement: Supplementary file 1 — Supplementary Materials [file 41385_2019_235_MOESM1_ESM.pdf]

Supplementary Materials (Barry et. al., 2019)

Supplementary Materials and Methods

Enterocyte extraction

Upon harvest 3.5 cm section of the terminal colon was cut lengthwise and incubated for 45 min at 37°C in 4 ml of enterocyte dissociation buffer (1x Hanks' balanced salt solution without Mg and Ca plus 10 mM HEPES, 1 mM EDTA, and 5 µl/ml 2- β-mercaptoethanol. Enterocytes were harvested by centrifugation at 4°C at 2,000 g, washed twice in PBS, and stored at -80°C until lysis.

Intrarectal administration of Sivelestat

C3H mice (Charles River Laboratories, Italy) were infected with *C. rodentium* by oral gavage. From 3 to 8 days post-inoculation (dpi) 50 mg/kg of Sivelestat sodium tetrahydrate (Selleck Chemicals) in 5 % DMSO/ 95% PBS or vehicle control was delivered intrarectally to mice under anesthesia (isoflurane). Mice received further Sivelestat on 10-11 dpi.

Sample processing and Histological Analysis

Histological analysis and immunostaining were performed as described in [1] using antibodies listed in Table S1. Images were acquired using a Zeiss AxioVision Z3 microscope, using an AxioCam MRm camera and processed using Zen 2.3 Blue Version (Carl Zeiss MicroImaging GmbH, Germany). For H&E images of entire colon sections, individual 40 x magnification images were taken as tiles and fused together using Zen. The number of Ly6G positive cells present in the mucosa region of a transverse cross section were manually counted using Zen 2.3 Blue software and data analysed using multiple-comparison one-way Analysis of Variance (ANOVA), Prism 7.

TUNEL Assay

TUNEL staining was performed using DeadEnd™ Fluorometric TUNEL System (Promega) as per manufacturer's instructions. DNA was counterstained with DAPI. Images were acquired as described above. Quantification was performed using Fiji, Image J. The DAPI channel was used to select the area to be quantified (mucosa region of a transverse cross section). TUNEL positive cells in this area were quantified by analysing the number of particles (threshold min: 0, max: 5140; pixels: 20-infinity and roundness 0-1). Counts obtained were analysed using multiple-comparison one-way Analysis of Variance (ANOVA), Prism 7 (Graphpad).

Quantitative real-time RT-PCR

RNA was isolated using RNeasy mini kit (Qiagen) and cDNA synthesised using SuperScript IV VILO Master Mix (ThermoFisher Scientific). Quantitative real-time (RT)-PCR reactions were performed using the FAM-MGB Taqman gene expression probe for SerpinA3N [Cat: #Mm00776439_m1] and SerpinA3M [Cat: #Mm07306477_m1] (ThermoFisher Scientific). Relative mRNA levels were calculated after normalisation to VIC-MGB Gapdh [Cat: #Mm99999915_g1] using the ΔCt method.

S100A8 and MPO ELISA

S100A8 concentration was determined using a DuoSet Mouse S100A8 or MPO ELISA kit (R&D Systems), according to the manufacturer's instructions. Data was analysed using multiple-comparison one-way Analysis of Variance (ANOVA), Prism 7.

Generation of neutrophil elastase substrate

80 mg of Rink Amide resin (0.04 mmol) was activated with 2 mL DCM for 30 min. The resin was filtered and washed with 1 mL DMF (4 x). The Fmoc-protecting group was removed from the resin by treatment with 20% piperidine (PIP) in DMF (3 x 1 mL) for 5 min, 5 min and 25 min. At this stage a ninhydrin test was performed to confirm the Fmoc deprotection reaction had proceeded to completion. Next, Fmoc-ACC-OH (44.1 mg, 0.1 mmol, 2.5 eq.), DICI (15.4 µL, 0.1 mmol, 2.5 eq.) and HOBt (13.5 mg, 0.1 mmol, 2.5 eq.) were dissolved in 1 mL of DMF and the mixture was gently shaken for 5 min. The mixture was then poured onto the resin and shaken for 24 h at RT. The resin was then filtered and washed with 1 ml of DMF (3 x), DCM (3 x) and DMF (3 x). A ninhydrin test was performed to check that the reaction had proceeded to completion. The Fmoc group was then removed using the same procedure described previously. Fmoc-Abu-OH (32. 5 mg, 0.1 mmol, 2.5 eq.), HATU (38 mg, 0.1 mmol, 2.5 eq.) and 2,4,6-trimethylpyridine (13.1 µL, 0.1 mmol, 2.5 eq.) were dissolved in 1 mL of DMF and gently agitated for 5 min. The mixture was then poured on the resin and the vessel was shaken for 24 h at RT. The resin was filtered and washed with 1 mL of DMF (3 x). The coupling was then repeated using half the amount of reagents. The Fmoc group was then removed using the same procedure. Next, Fmoc-Oic-OH (39 mg, 0.1 mmol, 2.5 eq.) was pre-activated with HOBt (15 mg, 0.1 mmol, 2.5 eq.) and DICI (14 µL, 0.1 mmol, 2.5 eq.) in 1 mL DMF and added to the resin, followed by gentle agitation for 1 h. A ninhydrin test was carried out after each coupling to check for reaction completion. The resin was filtered and washed with 1 mL of DMF (3 x), DCM (3 x) and DMF (3 x). The Fmoc-protecting group was removed using 20% PIP in DMF (3 x 2 mL) for 3 min per treatment. After the third treatment the resin was washed with 1 mL of DMF (3 x), DCM (3 x) and DMF (3 x). The same procedure was carried out

for coupling of Fmoc-Met(O2)-OH and Fmoc-Arg(NO_2)-OH. The peptide chain was then acetylated with AcOH (12 µL, 0.2 mmol, 5 eq.), HBTU (76 mg, 0.2 mmol, 5 eq.) and DIPEA (35 µL, 0.2 mmol, 5 eq.) in 1 mL DMF for 1 h. After 1 h, the resin was washed with 1 mL DMF (3 x), DCM (3 x), DMF (3 x), MeOH (3 x) and diethyl ether (3 x) and dried over P_2O_5 for at least 3 h. The peptide was cleaved from the resin by addition of 2 mL of TFA deprotection mixture (95 % TFA, 2.5 % TIS, 2.5 % H_2O) and gentle shaking for 2 h. The solution was collected in a 15 mL Falcon tube and the resin was washed once with 1 mL of TFA deprotection mixture. To the 3 mL of deprotection mixture was added 12 mL of cold diethyl ether. After precipitation (1 h, - 20°C) the mixture was centrifuged (5 min, 3000 rpm), washed with cold diethyl ether (4 mL), centrifuged again and left to dry at RT for 3 h. The solid was dissolved in DMSO:CH_3CN:H_2O (1:4.5:4.5) and purified by preparative LC-MS (Waters, column: X-Bridge C18, 5 µm particle size, 19 mm x 100 mm) using a CH_3CN:H_2O gradient. Pure fractions were lyophilized and the resulting solid was dissolved in dry DMSO to a concentrations of 10 mM and stored at −20°C until use.

Measurement of Kcat/Km

Substrates (Table S2) were serially diluted in FPEB and 25 µL was added to a 96-well plate. Next, 25 µL of mouse recombinant NE (4.6 nM for Ala-Ala-Pro-Val and 2.3 nm for Arg(NO_2)-Met(O2)-Oic-Abu) diluted in NE assay buffer (50 mM Tris, 1 M NaCl, 0.05%(w/v) Brij-35, pH 7.5) was added to each well containing the substrate. The enzyme-substrate reaction was monitored for 10 min using an Envision 2104 Multilabel Plate Reader in kinetic mode, with measurements taken using excitation/emission wavelengths of 355 nm/460 nm. The linear portion of each progress curve was used to calculate relative fluorescence units per second ($RFUs_{-1}$).

K_M, k_{cat}, and k_{cat}/K_M parameters were determined by nonlinear regression in Prism 7 (GraphPad Software). Each experiment was repeated three times and the results are presented as an average ± SEM.

Cloning and expression of recombinant Serpina3n

Serpina3n (amino acids 21-418) was PCR amplified from pCMV-SPORT6-Serpina3n (primers 1,2; Table S4), digested (*NcoI* and XhoI), ligated into pET-M11 (6x His) and transformed into *E. coli* Top10. The sequence verified construct was transformed into *E. coli* BL21 star and cells cultured in 100 ml Lysogeny broth (LB) with kanamycin, induced at OD 0.6 with 1mM IPTG at 20°C O/N. The cells were centrifuged (4550 g,15 min, 4°C) and pellet frozen at −80°C. Thawed cell pellets were re-suspended in lysis buffer (15ml of Tris-HCl 8.0, 250mM NaCl) and sonicated on ice followed by centrifugation (29,000 g, 20 min, 4 °C. The S/N were applied to pre-equilibrated in lysis buffer TALON His-Tag Purification Resin and incubated for 1 h rotating at 4 °C before applying to a gravity-flow column. The unbound proteins were washed with lysis buffer and bound fractions were eluted with 100mM imidazole in lysis buffer.

Generation of C. rodentium expressing His-tagged SerpinA3N-HlyA fusion

The amino acid sequence of SerpinA3N lacking signal peptide (amino acids 21-418) was synthesized (GeneArt, ThermoFisher Scientific) and cloned NcoI, NotI into pEHLYA5 (primers 3, 4). To generate pVDL-SerpinA3N the fragment between BglII site was cloned into pVDL9.3. The homology regions flanking the *xylE* locus were amplified (primers 5-8) and cloned into pSEVA612S generating pSEVA612S-XylE. T0 terminator and promoter PN25 were synthesized and cloned SphI-XbaI into pSEVA612S-XylE. *Serpina3n-HlyA,HlyB,HlyD* operon was cloned into this plasmid by

Gibson assembly (primers 9-12) and the final pSEVA612S-Serpina3n was conjugated into wildtype *C. rodentium*. *E.coli* 1047 pRK2013 (20µl) was incubated (2h, 37°C) with CC118-λpir pSEVA612S-SerpinA3N (20µl) on LB agar. 40µl of wildtype *C. rodentium* pACBSR were added (4h, 37°C) and conjugants were selected with appropriate antibiotics. Individual colonies were grown in LB+Sm+L-arabinose (0.4%) (8h, 37oC) and plated. Plasmids and genomic insertions were verified by sequencing.

HlyA secretion assay

Cultures of *E. coli* strain HB2151 carrying pVDL9.3-SerpinA3N (*hlyB, hlyD*) and the indicated pEHLYA5-derivative were grown overnight (O/N) at 30°C (170 rpm) in liquid LB+Cm. Next, bacteria were inoculated in fresh medium and grown at 37°C (170 rpm) until OD 600 nm reached 0.5. At this point, bacteria were induced with 1 mM isopropyl-1-thio-β-D-galactoside (IPTG) and further incubated for 6 h with agitation (100 rpm). For the purification of the secreted His-tagged SerpinA3N-HlyA fusion, supernatants from induced cultures were loaded onto chromatography columns with pre-equilibrated Cobalt-containing resin (Qiagen). Columns were washed with PBS buffer and e luted with the same buffer containing 150 mM imidazole. Fractions eluted from metal-affinity chromatography were dialysed against HEPES-buffer and concentrated in a 3 kDa centrifugal filter unit (Amicon Ultra-15, Merck). Protein concentration was estimated using QubitTM Protein Assay Kit (Thermo).

Growth curves

Saturated overnight cultures of bacterial strains were washed twice in M9 minimal media by centrifugation (2000 g, 5 minutes) and resuspension. Washed cultures were

diluted to an OD of 0.1 to a final volume of 100ul in M9 minimal media and OD_{600nm} measured in a plate reader (FLUOstar Omega, BMG Labtech) at 1 hr intervals at 37°C, shaking at 200rpm between measurements.

Supplementary Tables

Table S1: List of antibodies used in this study

**Antibody**	**Species**	**Concentration**	**Source**
SerpinA3N	Goat	Western blotting: 0.1 µg/ml	R&D Systems, AF4709
SLPI	Goat	Western blotting: 0.1 µg/ml	R&D Systems, AF1735
HSP90α	Mouse	Western blotting: 0.1 µg/ml	Santa Cruz, F-2
Ly6G	Rat	Immunofluorescence: 2.5 µg/ml	Biolegend, 1A8
Neutrophil Elastase/NE	Sheep	Immunofluorescence: 1 µg/ml	R&D Systems, AF4517
C. rodentium	Rabbit	1:200 dilution	[1]

Table S2: List of reagents used in this study

Reagent	Source
Mouse recombinant neutrophil elastase (NE)	R&D, 4517-SE
human NE	Enzo Life Sciences, SV-20927-01
Cathepsin C	Sigma, C8511
Dextran sulphate sodium (DSS; MW: 36,000-50,000)	MP Biomedicals
MeOSuc-Ala-Ala-Pro-Val-AMC substrate	Bachem, 4005227
MeOSuc-Arg(NO_2)-Met(O2)-Oic-Abu-ACC substrate	Generated in this study. The chemical structure provided in. Fig S3B.

Table S3: Disease activity index (DAI) for DSS

**SCORE**	**0**	**1**	**2**	**3**
**Weight loss**	<1%	1-5%	5-15%	>15%
**Stool consistency**	normal	loose	Very loose	Watery diarrhoea
**Hematochezia**	No blood	Visible blood on pellet	Gross bleeding	-

Table S4: List of primers used in this study

Number	Name	Sequence
1	rSerpinA3NpETFwd	CATGCCATGGGATTCCCAGATGGCACGTTGG
2	rSerpinA3NpETRev	CCGCTCGAGTCATTTGGGGTTGGCTATCTT
3	5-NcoI-SerpinA3N	ATGCCCATGGGACCAGATGGCACGTTGGGAATGG
4	3-NotI-SerpinA3N	CGATGCGGCCGCTTTGGGGTTGGCTATCTTGGC
5	5-HindIII-XylE-UP	ACTAGAAGCTTCTATTGCAGCAACCGCTTTG
6	3-SphI-XylE-UP	ACTTGCATGCACGCAGTACACCAGCAGCTG
7	5-SacI-XylE-DO	ACTTGAGCTCCCGTCTTTCAGCAGTTTGTC
8	3-EcoRI-XylE-DO	ACTTGAATTCAAGTAGTTTGCCAGCCACTG
9	5-GB-pVDL-Serp-I	TCCAAGAAGGAGATATACATATGGCTCATCACCATCACCA
10	3-GB-pVDL-Serp-I	ACGGGAGCTCGGTACCCGGGTTAACGCTCATGTAAACTTTCTG
11	5-GB-pVDL-Serp-P	AAAGTTTACATGAGCGTTAACCCGGGTACCGAGCTCCCGTCTT TCAGCAGTTTGTCGGCATTAACGTGG
12	3-GB-pVDL-Serp-P	TGGTGATGGTGATGAGCCATATGTATATCTCCTTCTTGGATCC GCGGCCGCTCTAGAAACTCC

Table S5: List of plasmids and strains used in this study

Strain/plasmid	Description	Reference
pCMV-SPORT6-*Serpina3n*	MGC Mouse Serpina3n cDNA in pCMV-SPORT6 vector	Dharmacon, CloneID: 4160649
pEHLYA5	ApR; pUC ori, lac promoter, N-terminal His tag, VHH, HA and E-tags, C-HlyA	2
pVDL9.3	CmR; expression of HlyB and HlyD	3
pVDL-Serpina3n	CmR; expression of HlyB and HlyD. Expresses Serpina3n inducible with IPTG	This study
pSEVA612S	GmR; R6K ori, oriT, I-SceI restriction sites flanking multicloning site	4
pSEVA612S-SerpinA3N	GmR; R6K ori, oriT, I-SceI restriction sites flanking XylE HR, T0-PN25-Serpina3n-HlyB-HlyD for constitutive secretion of Serpina3n	This study
pACBSR	SpR/SmR; p15A ori, PBAD, I-SceI endonuclease and λ-red genes inducible with 0.4% L-arabinose	5
DH10B-T1R	(F− λ−) mcrA Δmrr-hsdRMS-mcrBC φ80lacZΔM15, ΔlacX74, recA1, endA1, Δ(ara, leu)7697 galU galK rpsL(StrR), nupG tonA	Novagen-Merck
ICC169	Wild-type C. rodentium, O152 serotype, NalR	6
E. coli CC118-λpir	Δ(ara-leu) araD ΔlacX74 galE galK phoA20 thi- rpsE rpoB argE(Am) recA1, λpir	7
E. coli 1047 pRK2013	Helper strain for conjugation, KanR, oriColE1 RK2-Mob+ RK2-Tra+	8
HB2151	Δlac-pro, ara, nalR, thi, F'(proAB lacIQ lacZΔM15)	9

Supplementary References

1. Crepin, V. F., Collins, J. W., Habibzay, M. & Frankel, G. *Citrobacter rodentium* mouse model of bacterial infection. *Nat Protoc* **11**, 1851–1876 (2016).

2. Ruano-Gallego, D., Fraile, S., Gutierrez, C. & Fernández, L. Á. Screening and purification of nanobodies from *E. coli* culture supernatants using the hemolysin secretion system. *Microb. Cell Fact.* (2019).

3. Tzschaschel, B. D., Guzmán, C. A., Timmis, K. N. & Lorenzo, V. De An escherichia coli hemolysin transport system-based vector for the export of polypeptides: Export of shiga-like toxin iieb subunit by *Salmonella typhimurium aroa*. *Nat. Biotechnol.* (1996).

4. Martinez-Garcia, E., Aparicio, T., Goni-Moreno, A., Fraile, S. & Lorenzo, V. de SEVA 2.0: an update of the Standard European Vector Architecture for de-/re-construction of bacterial functionalities. *Nucleic Acids Res* **43**, D1183-9 (2015).

5. Herring, C. D., Glasner, J. D. & Blattner, F. R. Gene replacement without selection: Regulated suppression of amber mutations in *Escherichia coli*. *Gene* (2003).

6. Wiles, S. *et al.* Organ specificity, colonization and clearance dynamics in vivo following oral challenges with the murine pathogen *Citrobacter rodentium*. *Cell. Microbiol.* (2004).

7. Herrero, M., Lorenzo, V. de & Timmis, K. N. Transposon vectors containing non-antibiotic resistance selection markers for cloning and stable chromosomal insertion of foreign genes in gram-negative bacteria. *J. Bacteriol.* (1990).

8. Figurski, D. H. & Helinski, D. R. Replication of an origin-containing derivative of plasmid RK2 dependent on a plasmid function provided in trans. *Proc. Natl. Acad. Sci. U. S. A.* (1979).

9. Carter, P., Bedouelle, H. & Winter, G. Improved oligonudeotide site-directed rautagenesis using M13 vectors. *Nucleic Acids Res.* (1985).

Supplementary Figure legends

Figure S1: Colonic crypt hyperplasia in C57 and C3H mice. Crypt lengths measured from hematoxylin and eosin (H&E)-stained colon sections, n = 10. Kruskal-Wallis multiple comparisons test, *** $P \leq 0.001$.

Figure S2: Immunofluorescence staining of infected colon tissue sections. (**A**) Colon sections from infected C57 (8 dpi) and C3H (6 dpi) mice stained with Dapi (blue), Ly6G antibody (magenta) and neutrophil elastase (NE) antibody (white). 100x magnification, scale = 20µM. Arrows indicate neutrophils as determined by nuclear lobes (dapi) and Ly6G positivity. (**B**) NE staining of tissue sections from uninfected and infected mice. Top row DAPI (blue), NE (white) and *C. rodentium* (green). Bottom row is NE only. Scale = 20µM

Figure S3: Validation of NE substrates and recombinant SerpinA3N. (**A**) Samples from Fig. 2C incubated with the commercially available NE substrate (Ala-Ala-Pro-Val). A single time-point measurement taken at 2 hours is plotted as arbitrary fluorescence units (AFU) / 1000 for each individual sample. Multiple comparisons one-way ANOVA was performed *** $P \leq 0.001$. (**B**) Chemical structure of the Arg(NO_2)-Met(O2)-Oic-Abu substrate (**C**) k_{cat}/K_M plots of the commercial (Ala-Ala-Pro-Val) and optimised (Arg(NO_2)-Met(O2)-Oic-Abu) NE substrates. Results are presented as an average ± SEM, n=3. K_M, k_{cat}, and k_{cat}/K_M parameters as determined by nonlinear regression in GraphPad Prism 5. (**D**) Coomassie stain of recombinant SerpinA3N following TALEN purification. (**E)** Varying amounts of recombinant SerpinA3N or BSA was incubated with 5 nM recombinant NE for 30 min prior to the addition of NE substrate. Arbitrary fluorescence units (AFU) measured over time are plotted.

Figure S4: **Faecal NE activity reflects disease severity in DSS-induced colitis.** (**A**) Histological analysis of colonic sections of DSS-treated mice and representative images of TUNEL stained tissue sections, green: TUNEL, blue; Dapi. (**B**) Colon length measured on harvest, Multiple comparison one-way ANOVA, *** P ≤ 0.001. (**C**) Weight of mice presented as percentage of starting weight throughout DSS treatment, n = 8. (**D**) DAI based on sum of weight loss, diarrheal and hematochezia scores. (**E**) Levels of MPO in faeces from C57 mice treated with 1.5% and 3% DSS as determined by ELISA. Multiple comparison one-way ANOVA, * P ≤ 0.05.

Figure S5: SLPI is expressed by IECs but not detected in faecal samples. Western blot analysis of IECs and faecal samples from uninfected (UI) and infected (8 dpi) C57 mice using anti-SLPI and anti-SerpinA3N antibodies.

Figure S6: Calprotectin levels are significantly elevated during infection from day 4. Levels of S100A8 (calprotectin) in faeces from infected C3H mice over time as determined by ELISA. Multiple comparison one-way ANOVA, * P ≤ 0.05, ** P ≤ 0.01, *** P ≤ 0.001.

Figure S7: ICC2031 grows equivalent to wildtype *C. rodentium* in minimal media and does not affect colonic crypt hyperplasia *in vivo*. (**A**) Wildtype *C. rodentium* and ICC2031 were grown overnight in M9 minimal media and OD600 readings measured over time. Black line = wildtype, grey line = ICC2031. (**B**) Crypt lengths measured from hematoxylin and eosin (H&E)-stained colon sections. No significant difference (unpaired t test).

Figure S8: Intrarectal administration of Sivelestat reduces colitis severity in infected C3H mice. (**A**) Intrarectal (IR) administration of Sivelestat does not affect intestinal colonisation of *C. rodentium.* Colonisation as measured by CFU/gram of stool. Data are represented as mean ± SD, n ≥ 8. (**B**) Faecal NE activity in samples collect from mice 3 hrs post intrarectal injection of Sivelestat or vehicle control at 11 dpi. Mann-Whitney test. * P ≤ 0.05. (**C**) Diarrhoeal score of mice infected with *C. rodentium* and treated with Sivelestat or vehicle control over time; combined data from two biological repeats, n ≥ 8. Multiple t test, non-significant. (**D**) Survival curve of mice infected with *C. rodentium* and treated daily with Sivelestat or vehicle control.

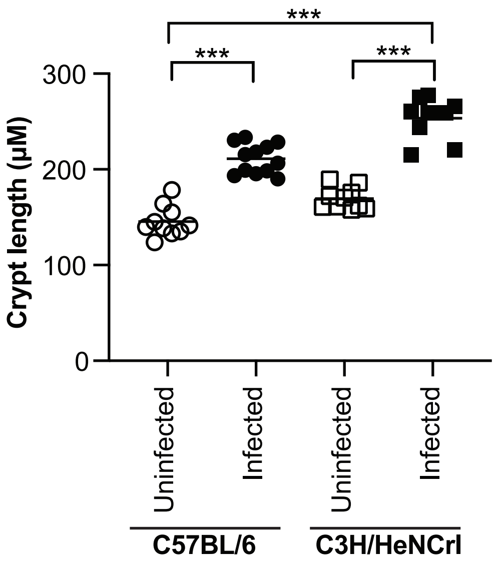
***
***
***
300
200
100
0
Crypt length (µM)
Uninfected
Infected
Uninfected
Infected
C57BL/6
C3H/HeNCrl

Figure S1

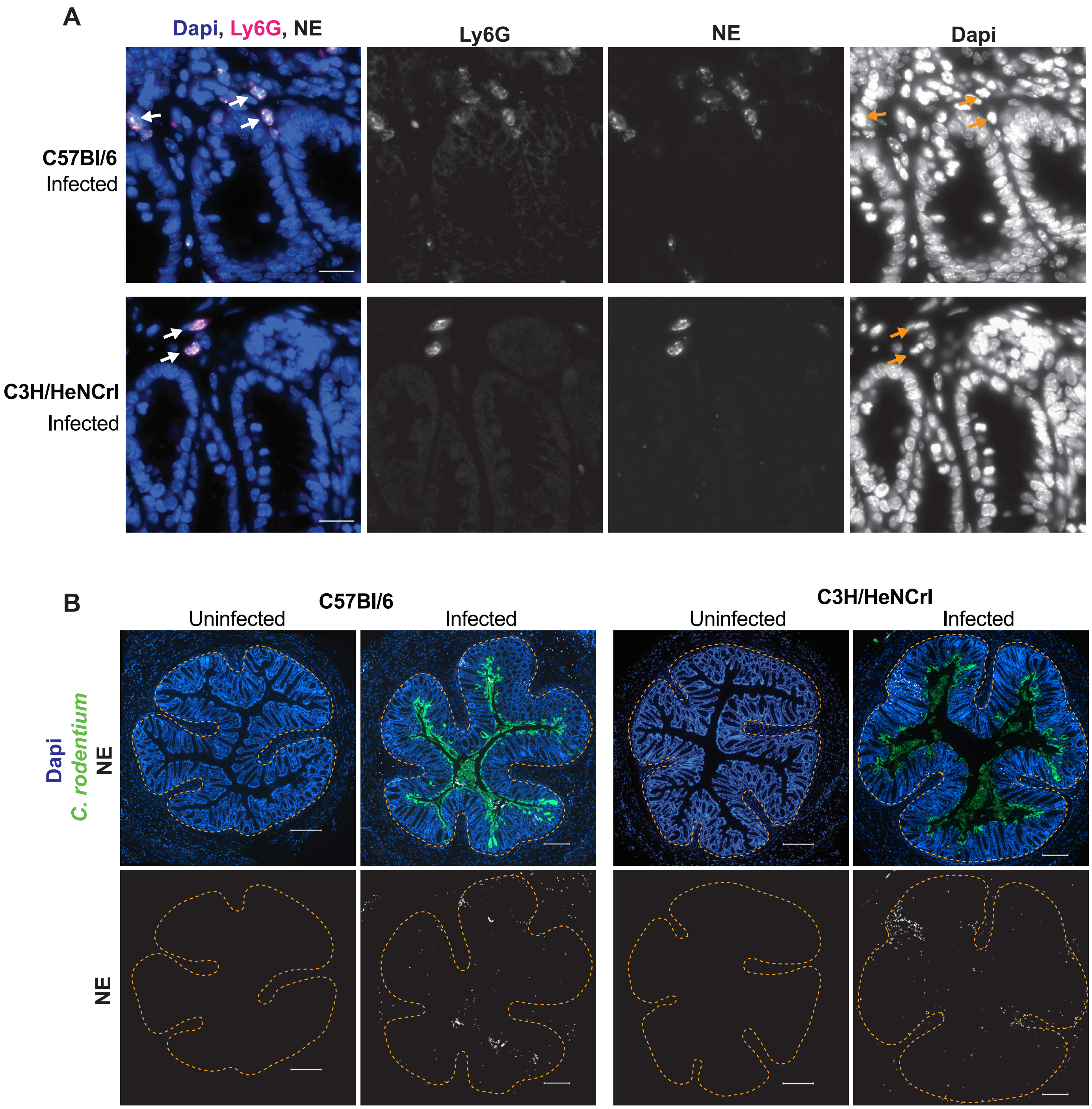
A
Dapi, Ly6G, NE
Ly6G
NE
Dapi
C57Bl/6
Infected
C3H/HeNCrl
Infected
B
C57Bl/6
Uninfected
Infected
C3H/HeNCrl
Uninfected
Infected
Dapi
C. rodentium
NE
NE

Figure S2

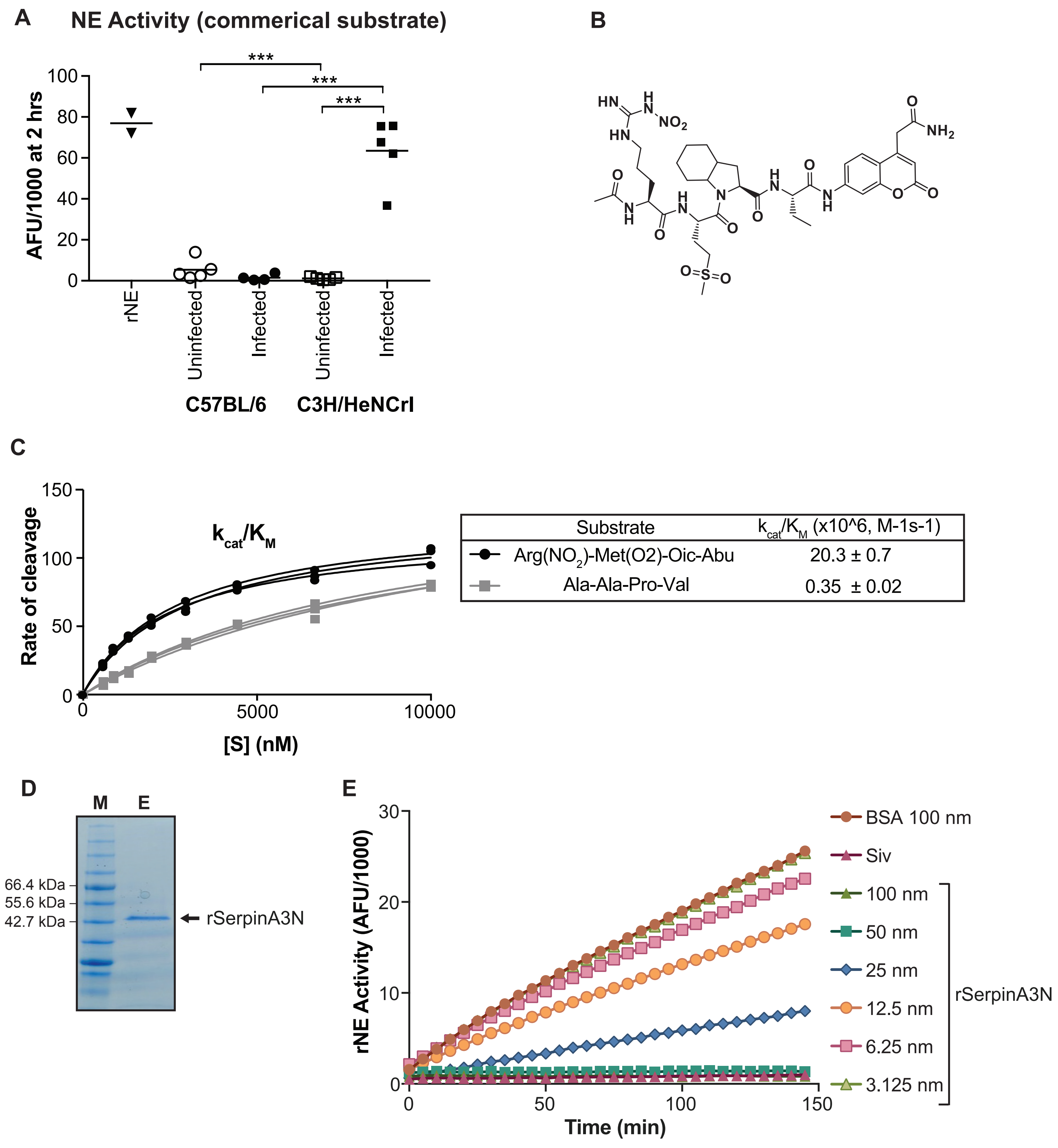
A
NE Activity (commerical substrate)
AFU/1000 at 2 hrs
100
80
60
40
20
0
***
***
***
rNE
Uninfected
Infected
Uninfected
Infected
C57BL/6
C3H/HeNCrl
B
HN
H
N
NO2
HN
O
NH2
O
N
H
O
N
H
H
N
O
N
O
O
H
N
O
N
H
O
O
O=S=O
C
Rate of cleavage
150
100
50
0
kcat/KM
0
5000
10000
[S] (nM)
Substrate
kcat/KM (x10^6, M-1s-1)
Arg(NO2)-Met(O2)-Oic-Abu
20.3 ± 0.7
Ala-Ala-Pro-Val
0.35 ± 0.02
D
M
E
66.4 kDa
55.6 kDa
42.7 kDa
rSerpinA3N
E
rNE Activity (AFU/1000)
30
20
10
0
0
50
100
150
Time (min)
BSA 100 nm
Siv
100 nm
50 nm
25 nm
12.5 nm
6.25 nm
3.125 nm
rSerpinA3N

Figure S3

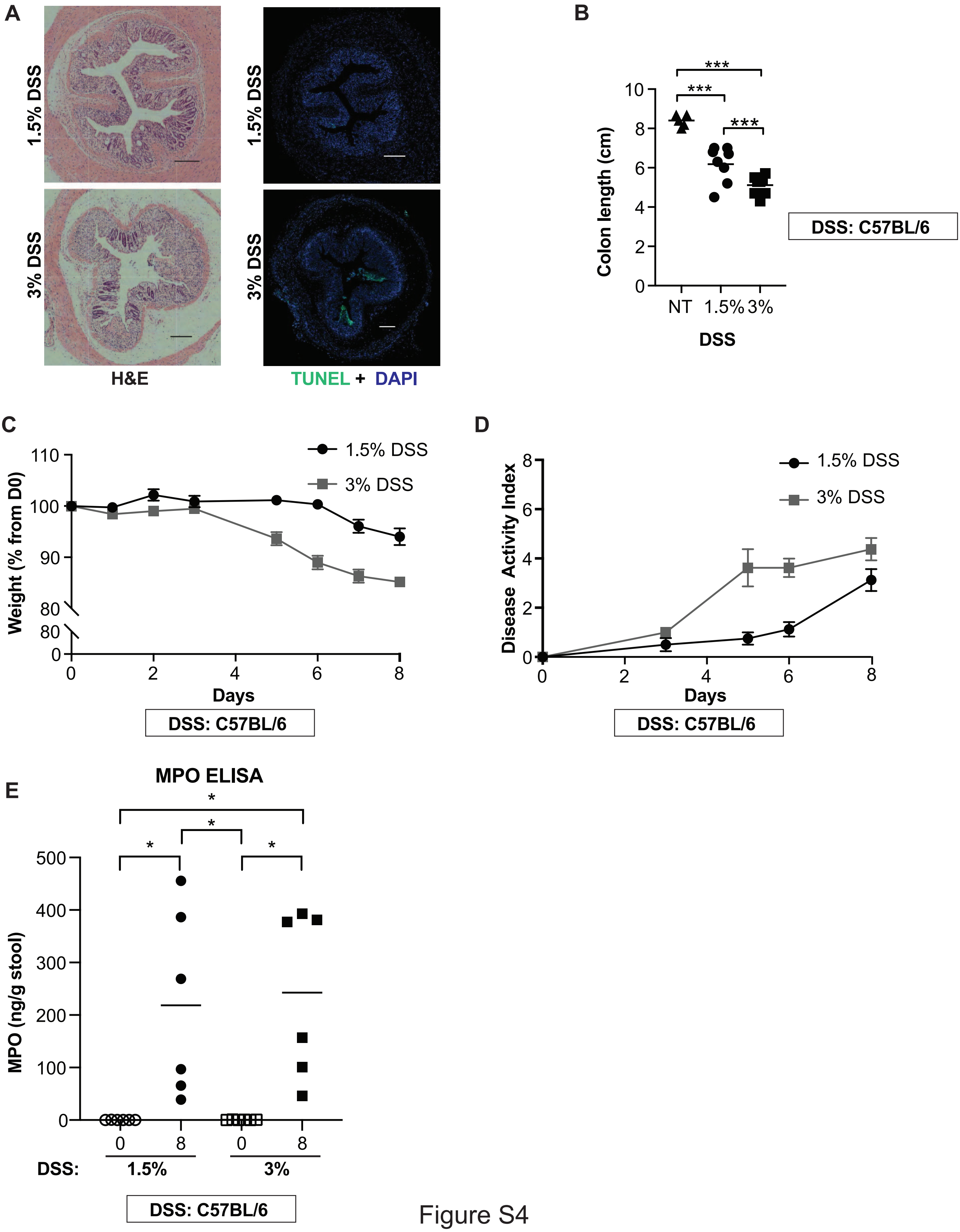
A
1.5% DSS
3% DSS
1.5% DSS
3% DSS
H&E
TUNEL + DAPI
B
***
***
***
Colon length (cm)
10
8
6
4
2
0
NT
1.5%
3%
DSS
DSS: C57BL/6
C
1.5% DSS
3% DSS
Weight (% from D0)
110
100
90
80
80
0
0
2
4
6
8
Days
DSS: C57BL/6
D
1.5% DSS
3% DSS
Disease Activity Index
8
6
4
2
0
0
2
4
6
8
Days
DSS: C57BL/6
E
MPO ELISA
*
*
*
*
MPO (ng/g stool)
500
400
300
200
100
0
0
8
0
8
DSS:
1.5%
3%
DSS: C57BL/6

Figure S4

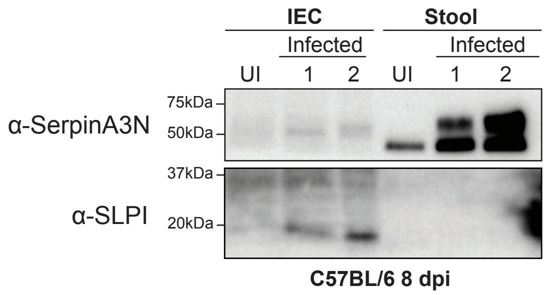
IEC
Stool
Infected
Infected
UI
1
2
UI
1
2
α-SerpinA3N
75kDa
50kDa
37kDa
α-SLPI
20kDa
C57BL/6 8 dpi

Figure S5

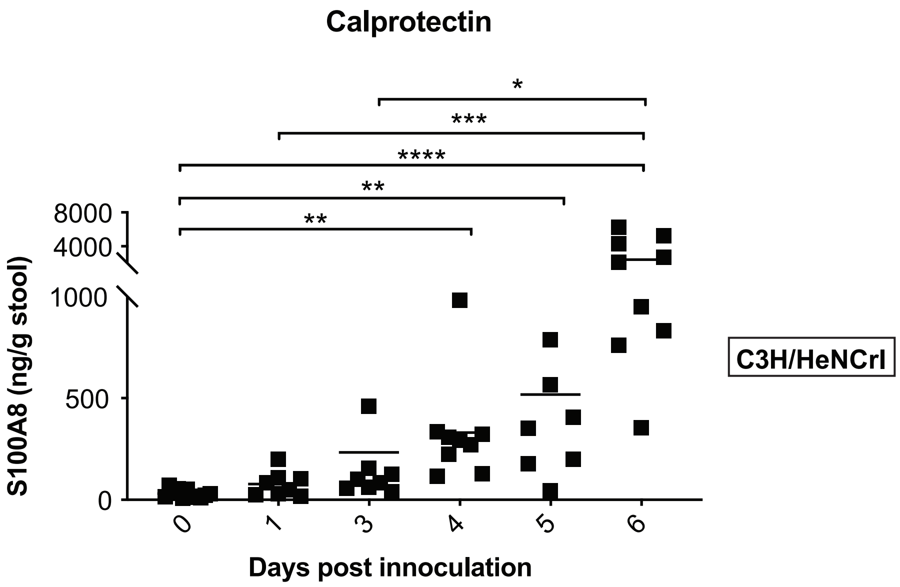
Calprotectin
*
***
****
**
**
8000
4000
1000
500
0
S100A8 (ng/g stool)
0
1
3
4
5
6
Days post innoculation
C3H/HeNCrl

Figure S6

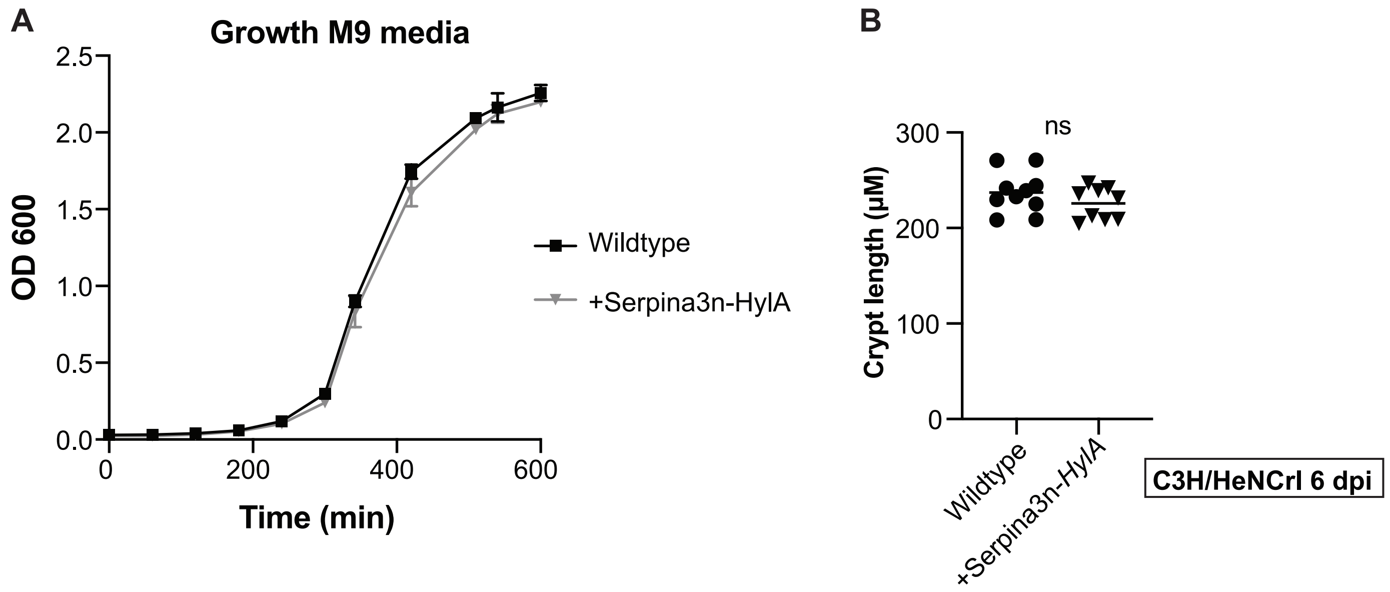
A
Growth M9 media
OD 600
2.5
2.0
1.5
1.0
0.5
0.0
0
200
400
600
Time (min)
Wildtype
+Serpina3n-HylA
B
ns
Crypt length (μM)
300
200
100
0
Wildtype
+Serpina3n-HylA
C3H/HeNCrl 6 dpi

Figure S7

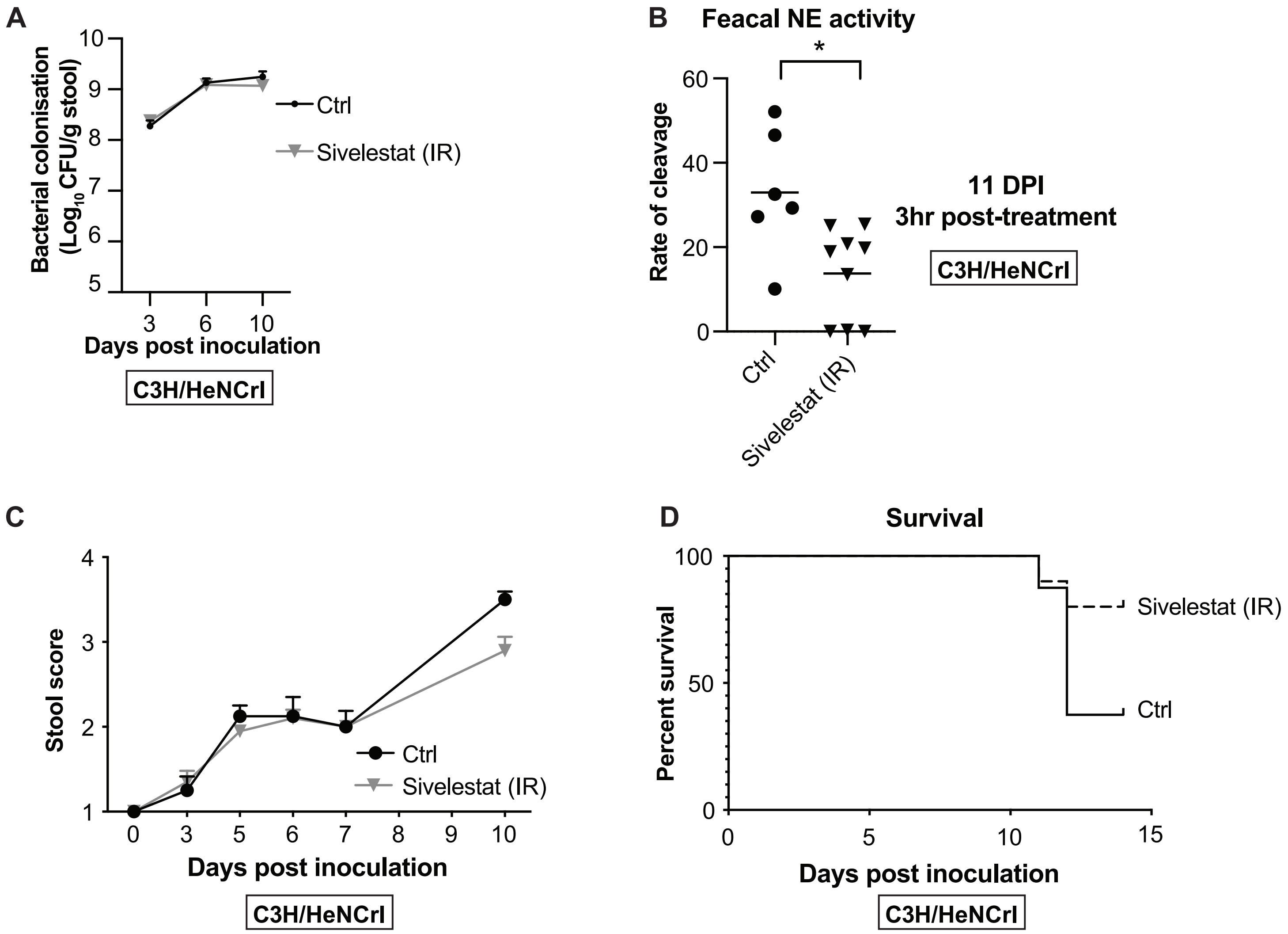
A
10
9
8
7
6
5
Bacterial colonisation (Log10 CFU/g stool)
3
6
10
Days post inoculation
Ctrl
Sivelestat (IR)
C3H/HeNCrl
B
Feacal NE activity
*
60
40
20
0
Rate of cleavage
Ctrl
Sivelestat (IR)
11 DPI
3hr post-treatment
C3H/HeNCrl
C
4
3
2
1
Stool score
0
3
5
6
7
8
9
10
Days post inoculation
Ctrl
Sivelestat (IR)
C3H/HeNCrl
D
Survival
100
50
0
Percent survival
0
5
10
15
Days post inoculation
Sivelestat (IR)
Ctrl
C3H/HeNCrl

Figure S8
